# New Boron Containing Acridines: Synthesis and Preliminary Biological Study

**DOI:** 10.3390/molecules28186636

**Published:** 2023-09-15

**Authors:** Anna A. Druzina, Nadezhda V. Dudarova, Ivan V. Ananyev, Anastasia A. Antonets, Dmitry N. Kaluzhny, Alexey A. Nazarov, Igor B. Sivaev, Vladimir I. Bregadze

**Affiliations:** 1 A.N. Nesmeyanov Institute of Organoelement Compounds, Russian Academy of Sciences, 28 Vavilov Str., 119334 Moscow, Russia; nadezjdino_96@mail.ru (N.V.D.); sivaev@ineos.ac.ru (I.B.S.); bre@ineos.ac.ru (V.I.B.); 2 N.S. Kurnakov Institute of General and Inorganic Chemistry, Russian Academy of Sciences, 31 Leninskii pr., 119991 Moscow, Russia; i.ananyev@gmail.com; 3 Department of Chemistry, M.V. Lomonosov Moscow State University, 1/3 Leninskie Gory, 119991 Moscow, Russia; antonets.anastasia.a@gmail.com (A.A.A.); nazarov@med.chem.msu.ru (A.A.N.); 4 V.A. Engelhardt Institute of Molecular Biology, Russian Academy of Sciences, 32 Vavilov Str., 11991 Moscow, Russia; uzhny@mail.ru; 5 Basic Department of Chemistry of Innovative Materials and Technologies, G.V. Plekhanov Russian University of Economics, 36 Stremyannyi Line, 117997 Moscow, Russia

**Keywords:** acridine, cobalt bis(dicarbollide), synthesis, DNA-interaction, antiproliferative activity

## Abstract

The synthesis of the first conjugates of acridine with cobalt bis(dicarbollide) are reported. A novel 9-azido derivative of acridine was prepared through the reaction of 9-methoxyacridine with N_3_CH_2_CH_2_NH_2_, and its solid-state molecular structure was determined via single-crystal X-ray diffraction. The azidoacridine was used in a copper (I)-catalyzed azide-alkyne cycloaddition reaction with cobalt bis(dicarbollide)-based terminal alkynes to give the target 1,2,3-triazoles. DNA interaction studies via absorbance spectroscopy showed the weak binding of the obtained conjugates with DNA. The antiproliferative activity (IC_50_) of the boronated conjugates against a series of human cell lines was evaluated through an MTT assay. The results suggested that acridine derivatives of cobalt bis(dicarbollide) might serve as a novel scaffold for the future development of new agents for boron neutron capture therapy (BNCT).

## 1. Introduction

Boron neutron capture therapy (BNCT) is a binary therapeutic method based on the nuclear capture reaction that takes place when the stable isotope ^10^B is irradiated with neutrons to release high-energy α-particles and ^7^Li nuclei [1,2]. The most important requirements for BNCT agents are (1) low toxicity; (2) high tumor uptake (~20–35 μg ^10^B) and low normal tissue uptake, with a sufficient tumor/normal tissue boron concentration ratio of >3:1; and (3) relatively rapid clearance from the blood and normal tissues, and persistence in the tumor for at least several hours during irradiation with a neutron flux [3,4,5,6]. Since α-particles have very short pathlengths in biological tissues (5–9 μm), their destructive effects are practically limited to cells that contain boron. In theory, α-particles can selectively destroy not only tumor cells, but also adjoining normal cells. Because BNCT primarily is a biologically, rather than physically, targeted type of particle radiation therapy, it offers the possibility to selectively destroy tumor cells without affecting the normal cells and tissues of an organism. The requirement, however, is that sufficient amounts of ^10^B and thermal neutrons are delivered to the site of the tumor.

Therefore, polyhedral boron hydrides, such as cobalt bis(dicarbollide) [3,3’-Co(1,2-C_2_B_9_H_11_)_2_]^−^, containing eighteen boron atoms in the molecule and characterized by outstanding chemical and thermal stability and the availability of convenient modification methods [7,8], are good candidates for the design of BNCT agents. Cobalt bis(dicarbollide) as a sodium salt demonstrates good water solubility and low toxicity both in vitro [9,10] and in vivo [9,11]. In particular, it does not show acute toxicity when intravenously [9] or intraperitoneally [11] injected into wild-type mice. It was also found that cobalt bis(dicarbollide) can pass directly through lipid membranes [12,13,14] and accumulate in cells without disrupting membrane integrity [10].

Calculations have shown that the radiobiological effectiveness of the boron neutron capture reaction [^10^B(^1^n,^4^He)^7^Li] can be significantly enhanced when it occurs in the cell nucleus rather than in the cytoplasm or the cell membrane [15]. Boron accumulating in the cell nucleus is much more efficient in cell killing than the same amount of boron when it is uniformly distributed. Consequently, when the BNCT drug is localized in the cell nucleus, a lower concentration is required [16]. These data have implications for the choice of boron carriers in neutron capture therapy.

This leads to an interest in DNA-binding BNCT agents, such as DNA intercalators (acridine, phenanthridinium, naphthalimide, and others) [17,18,19,20,21,22,23,24,25,26,27]. The first boron-containing acridine was synthesized by Snyder and Konecky and contained two aryl dihydroxyboryl groups [28]. This compound was too toxic to be used as a BNCT agent, but it led to the synthesis of the first carborane-based acridines [29]. Although these latter compounds were less toxic and achieved higher concentrations in tumors compared with the normal brain and blood, these values were significantly lower than those found in the liver, kidney, and spleen. Later, it was proposed to introduce additional hydrophilic groups into the carborane part of the molecule in order to improve its bioavailability [30]. Recently, synthesis of a series of acridines modified with carborane clusters and the evaluation of their DNA-binding ability and cytotoxicity has been described [31]. Also recently, one example of boronated acridine has been reported in which the boron moiety is cobalt bis(dicarbollide). This compound was synthesized through the reaction of 9-aminoacridine with a 1,4-dioxane derivative of cobalt bis(dicarbollide) [32].

In this contribution, we describe the synthesis of a series of novel 9-aminoacridine derivatives with the cobalt bis(dicarbollide) moiety as potential candidates for BNCT via the Cu(I)-catalyzed 1,3-dipolar [3 + 2] cycloaddition reaction of alkynes to azides (“click” reaction) as well as the evaluation of their antiproliferative activity and DNA interaction.

## 2. Results and Discussion

### 2.1. Synthesis of N^9^-Azidooacridine ***2***

9-Aminoacridine derivatives are an interesting group of heterocyclic compounds whose chemical structure causes them to have a variety of biological activities [33,34]. Thus, it was found that 9-aminoacridines can selectively accumulate in cell nuclei and other cellular organoids containing nucleic acids. Earlier studies on 9-aminoacridine have shown that this compound intercalates between the base stacks into a DNA double helix. It is assumed that the 9-aminoacridine with its 9-amino group lies in the minor groove and the 4- and 5-positions of the acridine ring are oriented toward the major groove [35]. It should also be noted that the literature provides information that the cytotoxicity of acridine derivatives is related to the presence and nature of various types of substituents at the 9-amino group of the acridine heterocycle. Thus, the substitution of one of the hydrogen atoms of the amino group in 9-aminoacridine leads to a decrease in the toxicity of substances [36].

We decided to synthesize boron-containing acridine derivatives with the introduction of the cobalt bis(dicarbollide) moiety through the “click” reaction. Among the methods for obtaining bioconjugates, the “click” reaction is widely used, leading to the formation of 1,2,3-triazoles [37,38,39]. Earlier, the “click” reaction was successfully used to obtain a wide range of conjugates of cobalt bis(dicarbollide) with chlorine *e*_6_ [40], nucleosides [41], and cholesterol [42]. In the present work, we used the “click” reaction to obtain new conjugates of the cobalt bis(dicarbollide) with acridine. At the start of our study, only a few examples of known boron-containing acridines were represented by carborane derivatives [29,31], while the first example of conjugate of acridine with cobalt bis(dicarbollide) was reported very recently, and it was prepared through the direct reaction of 9-aminoacridine with the 1,4-dioxane derivative of cobalt bis(dicarbollide) [32].

**Scheme 1:**
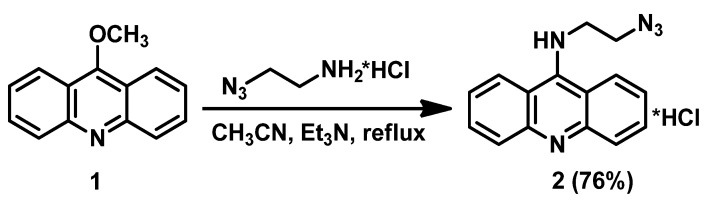
Synthesis of N^9^-substituted azidoacridine **2**.

Thus, we prepared the azido derivative of acridine which can be used for conjugation with acetylenic derivatives of cobalt bis(dicarbollide). The reaction of 9-methoxyacridine **1** with 2-azidoethanamine hydrochloride in acetonitrile in the presence of Et_3_N upon reflux for 17 h results in *N*^9^-azidoacridine hydrochloride **2**, which was isolated as a water-soluble pale yellow crystalline solid after crystallization from ethanol in 76% yield (Scheme 1).

The synthesized azido derivative of acridine **2** was characterized using the methods of ^1^H and ^13^C NMR spectroscopy, IR-spectroscopy, and high-resolution mass spectrometry (see Supplementary Materials). In the ^1^H-NMR spectrum, the signals of the methylene groups are observed at 3.63 and 3.72 ppm, and the characteristic signals of the acridine core **2** are observed in the range of 6.96–7.61 ppm. The IR spectrum of compound **2** exhibits an absorption band characteristic of the N_3_ group at 2065 cm^−1^.

### 2.2. Single-Crystal X-ray Diffraction Studies of N^9^-Azidoacridine ***2***

**Figure 1:**
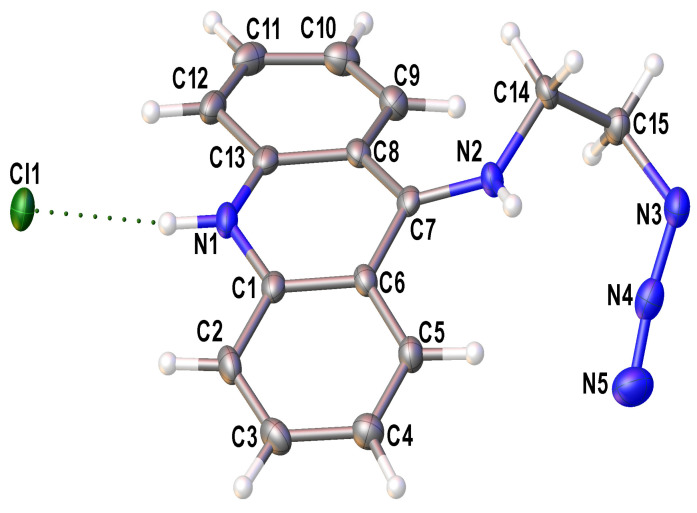
The independent unit in crystal of **2** in the representation of non-hydrogen atoms as probability ellipsoids of atomic displacements (*p* = 0.5). The H-bond is shown by a dotted line.

The solid-state structure of 9-azidoacridine **2** was determined through a single-crystal X-ray diffraction study (Figure 1). Crystals of **2** suitable for single-crystal X-ray analysis were grown via crystallization from ethanol.

The independent part of the unit cell of **2** contains an H-bonded contact ionic pair: the chloride anion and the protonated substituted acridine as a cation. According to a Cambridge Structural Database [43] search, the structure of **2** is one of many known examples of protonated acridine salts [44,45,46,47,48]. Note that in all these structures, the acridinium cation is nearly flat, while this is not the case for **2,** where the angle between mean-squared planes composed of the carbon atoms of two lateral hydrocarbon rings is 21.6 (2)°. Moreover, the N2 nitrogen atom in **2** is significantly shifted out of the plane of the central acridine cycle: the non-bonding N1…C7-N2 angle equals 167.0 (1)°. Considering the opposite directions of the displacement of the substituent and the lateral acridine cycles from the mean-square acridine plane, one can suppose the presence of steric repulsion between them. Indeed, there are several rather short H…H contacts within the cation; with the normalization of X-H bond lengths, the distances between the hydrogen atoms at the C5 and C9 atoms and the hydrogen atoms at the N2, C14, and C15 atoms are less than 2.05 Å.

The role of steric repulsion is supported by the geometry optimization performed for the isolated cation on the PBE0-D3/def2TZVP level. The relaxed structure is heavily distorted even without the influence of media effects: the mentioned interplane angle is 12.1° and the N1…C7-N2 angle equals 165.8°. Overall, both the relaxed isolated and crystal structures of cation **2** are quite similar (the r.m.s. difference for non-hydrogen atoms does not exceed 0.12 Å, Figure S1). What is noteworthy is that this conformation is indeed unfavorable for the acridine moiety: according to the calculations for the unsubstituted acridinium cation, the energy of the distorted conformation (constructed on the basis of that in **2** with the optimization of only hydrogen atoms) is 6.3 kcal·mol^−1^ higher than that in the fully relaxed structure. The electronic structure of the isolated cation **2** was then analyzed using the real space methods to determine the role of interatomic contacts in the (de)stabilization of the cation’s conformation.

**Figure 2:**
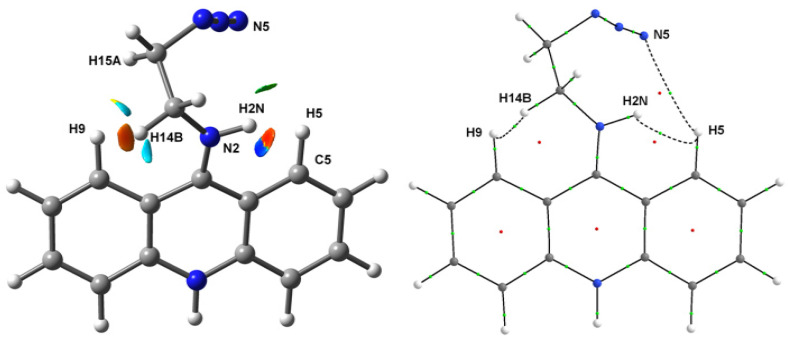
Real space electronic structure peculiarities in the area of substituent in the isolated optimized cation from **2**. On the left: the RDG isosurface (0.3) colored by the sign (*λ*_2_)·*ρ* (**r**) function (from −0.0019 a.u. in red to 0.0019 a.u. in blue). On the right: the atomic connectivity graph according to the topological analysis of *ρ* (**r**); dashed curves denote non-covalent interactions; green and red points denote (3, −1) and (3, +1) critical points of *ρ* (**r**), correspondingly.

According to the analysis of non-covalent interactions based on the reduced density gradient (RDG) and sign (*λ*_2_)·*ρ* (**r**) functions [49] (*λ*_2_—intermediate eigenvalue of electron density Hessian), there are a number of regions having low RDG values, which indicate the presence of non-covalent interactions in the area between the substituent and the acridine moiety. In particular, the RDG/sign (*λ*_2_)·*ρ* (**r**) plot (Figure S2) and the corresponding 3D isosurface plot (Figure 2, left) demonstrates the regions potentially having (1) rather strong non-covalent interactions between the H2N and H5 atoms and between the H9 and H14B atoms (the negative *λ*_2_ sign corresponds to the accumulation of electron density), (2) weak Van der Waals interaction between the N5 and H5 atoms, and (3) forced interactions between the H9 and H15A atoms, between the H9 and N2 atoms, and between the N2 and C5 atoms, all formed due to steric effects (the positive *λ*_2_ sign corresponds to the electronic charge depletion). The bonding nature of the dihydrogen H2N…H5 and H9…H14B contacts as well as of the weak N5…H5 contact is confirmed by the presence of (3, −1) critical points of electron density between corresponding topological atoms (Figure 2, right) that is the real space manifestation of preferred exchange–interaction channels [50,51]. Note that the net energy of these bonding non-covalent interactions estimated from the interatomic surface integrals of electron density [52] equals −7.1 kcal·mol^−1^, which is in exceptionally good agreement with the loss of energy occurring upon the distortion of acridine moiety conformation (see above). The main contribution (−3.6 and −3.2 kcal·mol^−1^) arises from the dihydrogen bonds, whereas the energy of C-H…N interaction is less than 0.3 kcal·mol^−1^ in magnitude. Thus, the steric repulsion between the substituent and acridine fragments leading to the pronounced distortion of the aromatic fragment is compensated by two dihydrogen bonds.

**Figure 3:**
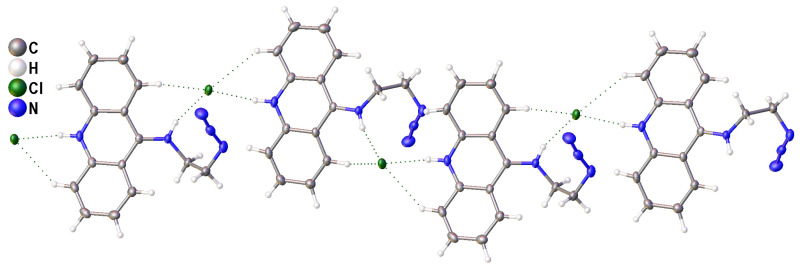
A fragment of infinite chain in the crystal of **2**. H-bonds are shown by dotted lines.

Finally, the crystal packing of **2** is expected, considering the presence of two strong proton donors in the cation. Namely, cation moieties are aggregated into the infinite chains by rather strong H-bonds between the N-H groups and chloride anions (N…Cl 3.131 and 3.156 Å, ∠ (NHCl) with the normalization of N-H bond lengths 171.3° and 156.4°, Figure 3). Due to their geometry, these H-bonds, together with weaker C-H…Cl contacts, can additionally stabilize the distorted structure of the cation. The abovementioned chains are bound together through numerous weaker interactions such as π…π stacking interactions between acridine moieties, C-H…Cl hydrogen bonds, and C-H…π interactions.

### 2.3. Synthesis of Cobalt Bis(dicarbollide)-Acridine Derivatives with 1,2,3-Triazoles ***7–10***

One of the important goals of BNCT, as already mentioned, is the synthesis of compounds which provide higher accumulation of boron in tumor than the clinically used compounds. This can be achieved through using boron clusters with a high content of boron atoms in the molecule, such as cobalt bis(dicarbollide) [6,53]. In this contribution, the “click” reaction was proposed to combine the cobalt bis(dicarbollide) cluster, providing a high content of boron atoms in the molecule, and the acridine system, providing the delivery of the boron component to the cell nucleus due to intercalation between DNA base stacks. Moreover, through changing the type and the size of a spacer between cobalt bis(dicarbollide) cluster and acridine, it is possible to control, to some extent, the hydrophilic/hydrophobic balance of the compounds.

**Scheme 2:**
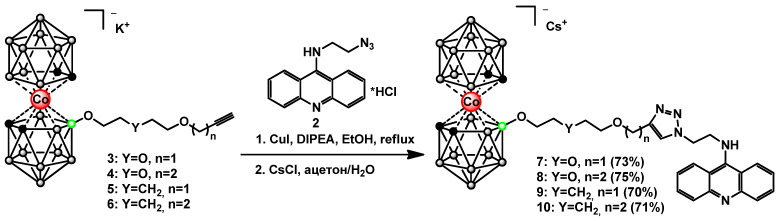
Synthesis of conjugates of cobalt bis(dicarbollide) with acridine **7–10**.

The obtained *N*^9^-azidooacridine **2** was used for the synthesis of target conjugates of cobalt bis(dicarbollide) with acridine **7**–**10** (Scheme 2). The acetylenic derivatives of cobalt bis(dicarbollide) **3**–**6** were prepared through the nucleophilic cleavage reactions of the corresponding cyclic oxonium derivatives of cobalt bis(dicarbollide) with 2-propyn-1-ol and 3-butyn-1-ol [40,54]. The “click” reactions were carried out refluxing boron-containing acetylenic derivatives **3**–**6** with the *N*^9^-azidoacridine **2** in ethanol for 2 h in the presence of CuI and diisopropylethylamine (DIPEA). The desired triazoles **7**–**10** were isolated as cesium salts via precipitation with CsCl from aqueous acetone followed by column chromatography on silica using a CH_2_Cl_2_-CH_3_CN mixture (70–75%) as eluent.

The conjugation products were characterized via ^1^H-, ^11^B-, and ^13^C-NMR; IR-spectroscopy; and high-resolution mass spectrometry (see Supplementary Materials). The purity of the obtained compounds was confirmed via chemical analysis.

In the ^1^H-NMR spectra of the synthesized compounds **7**–**10**, the characteristic signals of the triazole C*H* hydrogens appear in the region of 7.83–7.99 ppm; the signals of the methylene groups next to the triazole cycle are observed in the range of 4.90–5.14 ppm and the characteristic signals of the aromatic system of acridine are observed in the range of 7.62–8.58 ppm for compounds **7**–**10**. For conjugates **7**–**10**, the signals of the C*H*_carb_ groups in the ^1^H-NMR spectra appear as broad singlets in the ranges of 4.19–4.25 and 4.11–4.19 ppm. In the ^13^C-NMR spectra, the signals of the triazole *C*H carbons for **7**–**10** are observed in the range of 123.2–124.0 ppm, whereas the signals of the second triazole carbons appear in the range of 139.8–140.2 ppm. In the ^13^C-NMR spectra, the signals of *C*H_carb_ groups appeared in the range of 46.4–53.9 ppm. The ^11^B{^1^H} NMR spectra of compounds **7**–**10** exhibit singlets between 23.4–23.8 ppm from the substituted boron atom.

**Figure 4:**
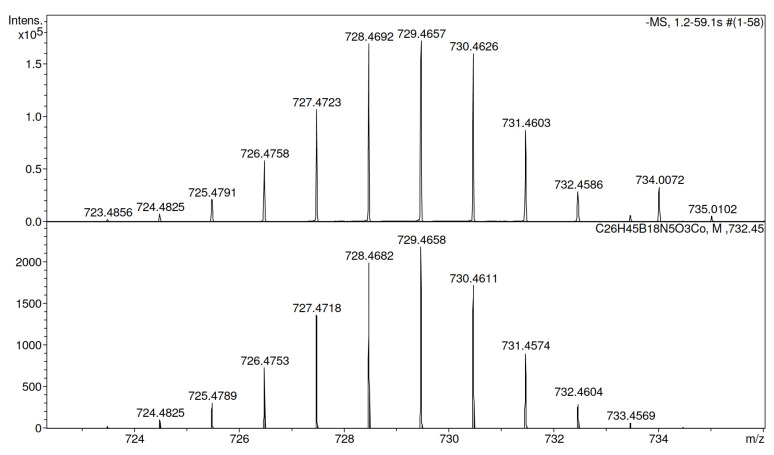
Mass spectrum of conjugate **7** (found 729.4657 [M]^−^, [C_26_H_45_B_18_CoN_5_O_3_]^−^, calculated 729.4658).

The IR spectra of compounds **7**–**10** exhibit absorption bands characteristic of the BH groups at 2547–2553 cm^−1^ and the 1,2,3-triazole heterocycles at 1581–1593 cm^−1^. The mass spectra of boron-containing conjugates **7**–**10** show negatively charged ions corresponding to [M-Cs]^−^, which are consistent with the predicted isotope distributions; this confirms the molecular formulas of the obtained compounds (Figure 4 presents the mass spectrum of conjugate **7**).

### 2.4. DNA Interaction Study

**Figure 5:**
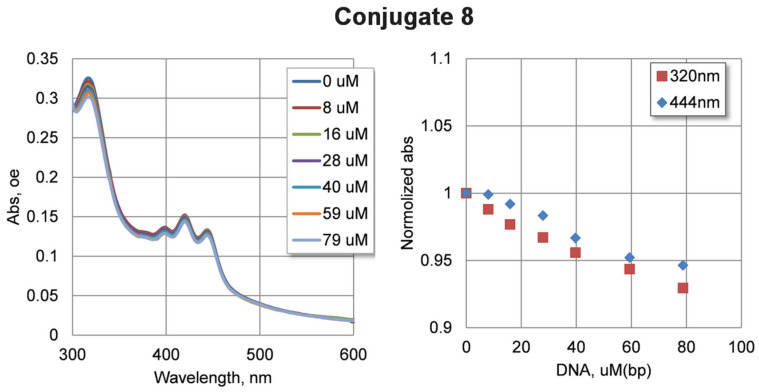
Changes of absorbance spectra upon DNA interaction for conjugate **8**. We used a 5 mkM compound in 10 mM potassium phosphate buffer pH = 8.0 and a calf thymus DNA in the range 0–80 µM (b.p.).

In order to test the ability of the compounds to bind DNA, spectral changes were tested through increasing the concentration of DNA in solution. Due to the low solubility of the compounds, a concentration of 5 µM was used. Calf thymus DNA at concentrations of up to 80 µM was added to the solution of compounds. The changes in the absorption spectra of compounds **7**–**10** are shown in Figure S5 (see Supplementary Materials). It can be seen that increasing the concentration of DNA in the solution leads to a non-significant decrease in absorbance. Changes in the absorbance of compounds **7**–**10** were not significant: about 5% taking into account dilution during titration. Such changes may characterize the relatively weak binding of the studied compounds with DNA under experimental conditions. The largest spectral changes were observed for conjugate **8** (Figure 5).

### 2.5. Antiproliferative Activity of Boronated Acridines ***7**–**10***

The antiproliferative activity of compounds **7**–**10** against cancer HCT116, MCF7, A549, and WI38 nonmalignant lung fibroblast cell lines was evaluated by means of a standard MTT colorimetric assay after 72 h of incubation (Table 1). All four compounds were found to be nontoxic against the lung cancer A549 cell line. However, against other cancer cells and nonmalignant cells, compounds showed activity in the mid-micromolar range that complicates use as new agents for BNCT.

## 3. Materials and Methods

### 3.1. General Methods

The acetylenic derivatives of cobalt bis(dicarbollide) [8-HC≡CCH_2_O(CH_2_CH_2_O)_2_-3,3′-Co(1,2-C_2_B_9_H_10_)(1′,2′-C_2_B_9_H_11_)]K (**3**) [40], [8-HC≡CCH_2_CH_2_O(CH_2_CH_2_O)_2_-3,3′-Co(1,2-C_2_B_9_H_10_)(1′,2′-C_2_B_9_H_11_)]K (**4**) [40], [8-HC≡CCH_2_O(CH_2_)_5_O-3,3′-Co(1,2-C_2_B_9_H_10_)(1′,2′-C_2_B_9_H_11_)]Na (**5**) [54], [8-HC≡CCH_2_CH_2_O(CH_2_)_5_O-3,3′-Co(1,2-C_2_B_9_H_10_)(1′,2′-C_2_B_9_H_11_)]Na (**6**) [54], and N_3_CH_2_CH_2_NH_2_ × HCl [56] were prepared according to the literature. 9-Methoxyacridine (Chemieliva Pharmaceutical Co., Ltd., Chongqing, China), diisopropylethylamine (Carl Roth GmbH, Karlsruhe, Germany), and CuI (PANREAC QUIMICA SA, Barcelona, Spain) were used without further purification. Ethanol, CH_3_CN, CH_2_Cl_2_, and NaN_3_ were commercially analytical grade reagents. The reaction progress was monitored via thin-layer chromatography (Merck F245 silica gel on aluminum plates). Acros Organics silica gel (0.060–0.200 mm) was used for column chromatography. The NMR spectra at ^1^H (400.1 MHz), ^11^B (128.4 MHz), and ^13^C (100.0 MHz) were recorded with a Varian Inova 400 spectrometer (Varian, Palo Alto, CA, USA). The residual signal of the NMR solvent relative to Me_4_Si was taken as the internal reference for ^1^H- and ^13^C-NMR spectra. ^11^B-NMR spectra were referenced using BF_3_∙Et_2_O as an external standard. Infrared spectra were recorded on a Spectra SF 2000 (OKB SPECTRUM, Saint-Petersburg, Russia) instrument. High-resolution mass spectra (HRMS) were measured on a mictOTOF II (Bruker Daltonic, Bremen, Germany) instrument using electrospray ionization (ESI). The measurements were performed in a negative ion mode (interface capillary voltage 3000 V) and positive ion mode (interface capillary voltage 4500 V), mass range from *m*/*z* 50 to *m*/*z* 3000, external or internal calibration was carried out with ESI Tuning Mix, Agilent. A syringe injection was used for solutions in acetonitrile (flow rate 3 µL/min). Nitrogen was applied as a dry gas; the interface temperature was set at 180 °C. Elemental analyses were performed at the Laboratory of Microanalysis of the A.N. Nesmeyanov Institute of Organoelement Compounds.

#### 3.1.1. Synthesis of N^9^-Azidoacridine ***2***

We added 1 mL of NEt_3_ to a suspension of 9-methoxyacridine **1** (0.5 g, 2.4 mmol) and 2-azidoethanamine hydrochloride (0.32 g, 2.6 mmol) in 20 mL of CH_3_CN. The reaction mixture was stirred under reflux for 15 h and then cooled to room temperature. To the cooling reaction mixture, a few drops of HCl were added before pH = 3 and left in the air for 1 h. Then, the solution was evaporated, and residue was crystallized from EtOH. The product was filtered, washed with cold EtOH (5 mL), and air dried to give pale yellow crystals of **2** (0.54 g, yield 76%). ^1^H NMR (400 MHz, D_2_O) д 7.61 (d, 2H, 2 × CH_Ar,_ J = 9.0 Hz), 7.49 (t, 2H, 2 × CH_Ar_), 7.11(t, 2H, 2 × CH_Ar_), 6.96 (d, 2H, 2 × CH_Ar_, J = 9.0 Hz), 3.72 (CH_2_), 3.63 (CH_2_) ppm. ^13^C NMR (101 MHz, D_2_O): 158.9 (C_Ar_), 138.6 (C_Ar_), 135.3 (CH_Ar_), 124.0 (2 × CH_Ar_), 118.0 (CH_Ar_), 111.5 (C_Ar_), 57.4 (C_Ar_), 49.7 (NCH_2_), 47.6 (NCH_2_) ppm. IR (KBr, н, cm*^−^*^1^): 2065 (N_3_). HRMS (ESI) *m*/*z* for [C_15_H_13_N_5_]^+^ calcd 264.1244 [M]^+^, found: 264.1246 [M]^+^.

##### General Procedure for the Synthesis of the Conjugates of Cobalt Bis(Dicarbollide) with Acridine **7**–**10**

A mixture of 9-azidoacridine **2** (1 eq.), the alkynyl derivatives of cobalt bis(dicarbollide) **3**–**6** (1 eq.), diisopropylethylamine (0.5–1 mL), and CuI (0.1 eq.) in 10–15 mL of EtOH was heated under reflux for 2 h. Then, the reaction mixture was cooled to room temperature, and inorganic precipitate was filtered. Then, organic solvent was removed in vacuo. The residue was extracted using EtOAc (100 mL) and washed with 1M HCl (4 × 50 mL) and brine (2 × 50 mL) and dried (Na_2_SO_4_). Then, the ethyl acetate was evaporated. The residue was dissolved in 5 mL of acetone. To the resulting solution, 1 g of CsCl in 100 mL of water was added. The crude product was purified on a silica column using CH_2_Cl_2_-CH_3_CN (2/1) as an eluent to give the desired products **7**–**10**.

#### 3.1.2. Synthesis of Conjugate **7**

Conjugate 7 was prepared from compound **2** (0.09 g, 0.30 mmol), the alkynyl derivative of cobalt bis(dicarbollide) **3** (0.14 g, 0.30 mmol), diisopropylethylamine (1 mL, 0.74 g, 5.73 mmol), and CuI (0.006 g, 0.03 mmol) in 15 mL of EtOH. The product was obtained as an orange solid of **4** (0.19 g, yield 73%). ^1^H NMR (400 MHz, acetone-d_6_) д 8.52 (d, 2H, 2 × C*H*_Ar_, *J* = 9.0 Hz), 8.02 (s, 4H, 4 × C*H*_Ar_), 7.83 (s, 1H, C*H*CN_3_), 7.62 (m, 2H, 2 × C*H*_Ar_), 5.08 (s, 2H, C*H*_2_N), 4.90 (s, 2H, C*H*_2_NH), 4.41 (s, 2H, OC*H*_2_C), 4.19 (br. s, 2H, C*H*_carb_), 4.11 (br. s, 2H, C*H*_carb_), 3.78 (s, 2H, BOC*H*_2_), 3.57 (s, 2H, C*H*_2_O), 3.49 (s, 2H, C*H*_2_O), 3.22 (s, 2H, C*H*_2_O) ppm. ^11^B NMR (128 MHz, acetone-d_6_): 23.8 (1B, s), 5.2 (1B, d, *J* = 144 Hz), 0.3 (1B, d, *J* = 152), −2.3 (1B, d, *J* = 164 Hz), −4.5 (2B, d, *J* = 142 Hz), −7.2 (4B, d, *J* = 124 Hz), −9.0 (2B, d, *J* unsolved), −17.3 (2B, d, *J* = 164 Hz), −20.2 (2B, d, *J* = 150 Hz), −22.5 (1B, d, *J* = 150 Hz), −28.6 (1B, d, *J* = 142 Hz) ppm. ^13^C NMR (101 MHz, acetone-d_6_): 158.3 (*C*_Ar_), 144.8 (*C*_Ar_), 140.2 (*C*N_3_CH), 134.9 (*C*H_Ar_), 125.1 (*C*H_Ar_), 123.9 (*C*H_Ar,_ CN_3_*C*H), 119.9 (*C*H_Ar_), 113.5 (*C*_Ar_), 72.2 (O*C*H_2_), 70.3 (O*C*H_2_), 68.8 (O*C*H_2_), 63.8 (O*C*H_2_), 59.7 (O*C*H_2_), 53.3 (*C*H_carb_), 50.0 (N*C*H_2_), 49.6 (N*C*H_2_), 46.6 (*C*H_carb_) ppm. IR (KBr, н, cm^−1^): 2568 (BH), 1581 (triazole). Found: C 36.41, H 5.14, B 22.79, N 8.07; Calc. for C_26_H_45_B_18_CoN_5_O_3_Cs C 36.22, H 5.26, B 22.57, N 8.12. HRMS (ESI) *m*/*z* for [C_26_H_45_B_18_CoN_5_O_3_]^-^ calcd 729.4658 [M]^−^, found 729.4657 [M]^−^.

#### 3.1.3. Synthesis of Conjugate **8**

Conjugate 8 was prepared from compound **2** (0.062 g, 0.21 mmol), alkynyl derivative of cobalt bis(dicarbollide) **4** (0.10 g, 0.21 mmol), diisopropylethylamine (0.5 mL, 0.37 g, 2.86 mmol), and CuI (0.004 g, 0.02 mmol) in 10 mL of EtOH. The product was obtained as an orange solid of **4** (0.14 g, yield 75%). ^1^H NMR (400 MHz, acetone-d_6_) д 8.54 (d, 2H, 2 × CH_Ar,_ J = 9.0 Hz), 8.02 (m, 4H, 4 × CH_Ar_), 7.87 (s, 1H, CHCN_3_), 7.66 (m, 2H, 2 × CH_Ar_), 5.08 (m, 2H, *C*H_2_N), 4.90 (m, 2H, *C*H_2_NH), 4.22 (br. s, 2H, *C*H_carb_), 4.19 (br. s, 2H, *C*H_carb_), 3.67 (m, 2H, O*C*H_2_CH_2_C), 3.48 (m, 8H, B*OC*H_2,_ 3 × CH_2_O), 2.80 (m, 2H, CH_2_O) ppm. ^11^*B* NMR (128 MHz, acetone-d_6_): 23.4 (1B, s), 4.4 (1*B*, d, J = 145 Hz), 0.6 (1*B*, d, J = 168), −2.4 (1*B*, d, J unsolved), −4.3 (2*B*, d, J = 156 Hz), −7.2 (2*B*, d, J = 130 Hz), −8.0 (4*B*, d, J = 124 Hz), −17.4 (2*B*, d, J unsolved), −20.4 (2*B*, d, J = 162 Hz), −21.9 (1*B*, d, J = 160 Hz), −28.7 (1*B*, d, J = 152 Hz) ppm. ^13^C NMR (101 MHz, acetone-d_6_): 158.6 (C_Ar_), 145.6 (C_Ar_), 139.9 (CN_3_CH), 135.6 (CH_Ar_), 125.2 (CH_Ar_), 124.3 (CH_Ar_), 123.4 (CN_3_CH), 119.2 (CH_Ar_), 112.9 (C_Ar_), 71.9 (OCH_2_), 70.2 (OCH_2_), 69.8 (OCH_2_), 69.4 (OCH_2_), 68.6 (OCH_2_), 53.7 (CH_carb_), 49.5 (NCH_2_), 48.8 (NCH_2_), 46.5 (CH_carb_), 29.5 (CH_2_) ppm. IR (KBr, н, cm*^−^*^1^): 2553 (BH), 1593 (triazole). Found: C 37.17, H 5.28, B 22.51, N 7.68; Calc. for C_27_H_47_B_18_CoN_5_O_3_Cs C 37.01, H 5.41, B 22.21, N 7.99. HRMS (ESI) *m*/*z* for [C_27_H_47_B_18_CoN_5_O_3_]^-^ calcd 743.4815 [M]^−^, found: 743.4818 [M]^−^.

#### 3.1.4. Synthesis of Conjugate **9**

Conjugate 9 was prepared from compound **2** (0.09 g, 0.30 mmol), the alkynyl derivative of cobalt bis(dicarbollide) **5** (0.14 g, 0.30 mmol), diisopropylethylamine (1 mL, 0.74 g, 5.73 mmol), and CuI (0.006 g, 0.03 mmol) in 15 mL of EtOH. The product was obtained as an orange solid of **4** (0.18 g, yield 70%). ^1^H NMR (400 MHz, acetone-d_6_) д 8.58 (d, 2H, 2 × CH_Ar,_ J = 9.0 Hz), 8.07 (m, 2H, 2 × CH_Ar_), 7.99 (m, 3H, 2 × CH_Ar_, CHCN_3_), 7.67 (t, 2H, 2 × CH_Ar_), 5.14 (m, 2H, *C*H_2_N), 4.96 (m, 2H, *C*H_2_NH), 4.46 (s, 2H, O*C*H_2_C), 4.25 (br. s, 2H, *C*H_carb_), 4.17 (br. s, 2H, *C*H_carb_), 3.46 (t, 2H, B*OC*H_2_), 3.33 (t, 2H, CH_2_), 1.44 (dq, 4H, 2 × CH_2_), 1.31 (m, 2H, CH_2_) ppm. ^11^*B* NMR (128 MHz, acetone-d_6_): 23.4 (1B, s), 4.3 (1*B*, d, J = 144 Hz), 0.0 (1*B*, d, J = 152), −2.6 (1*B*, d, J unsolved), −4.6 (2*B*, d, J = 162 Hz), −7.4 (2*B*, d, J = 122 Hz), −8.2 (4*B*, d, J = 124 Hz), −17.4 (2*B*, d, J = 162 Hz), −20.3 (2*B*, d, J = 144 Hz), −22.6 (1*B*, d, J = 158 Hz), −28.4 (1*B*, d, J = 150 Hz) ppm. ^13^C NMR (101 MHz, acetone-d_6_): 158.8 (C_Ar_), 145.6 (C_Ar_), 139.8 (CN_3_CH), 135.8 (CH_Ar_), 125.3 (CH_Ar_), 124.4 (CH_Ar_), 124.0 (CN_3_CH), 119.0 (CH_Ar_), 112.9 (C_Ar_), 70.0 (OCH_2_), 68.8 (OCH_2_), 63.8 (OCH_2_), 53.9 (CH_carb_), 49.3 (NCH_2_), 48.9 (NCH_2_), 46.4 (CH_carb_), 31.6 (CH_2_), 29.6 (CH_2_), 22.7 (CH_2_) ppm. IR (KBr, н, cm*^−^*^1^): 2553 (BH), 1583 (triazole). Found: C 37.83, H 5.32, B 22.91, N 8.33; Calc. for C_27_H_47_B_18_CoN_5_O_2_Cs C 37.70, H 5.51, B 22.62, N 8.14. HRMS (ESI) *m*/*z* for [C_27_H_47_B_18_CoN_5_O_2_]^-^ calcd 727.4866 [M]^−^, found: 727.4868 [M]^−^.

#### 3.1.5. Synthesis of Conjugate **10**

Conjugate 10 was prepared from compound **2** (0.063 g, 0.21 mmol), the alkynyl derivative of cobalt bis(dicarbollide) **6** (0.10 g, 0.21 mmol), diisopropylethylamine (0.5 mL, 0.37 g, 2.86 mmol), and CuI (0.004 g, 0.02 mmol) in 10 mL of EtOH. The product was obtained as an orange solid of **4** (0.13 g, yield 71%). ^1^H NMR (400 MHz, acetone-d_6_): 8.56 (d, 2H, 2 × CH_Ar,_ J = 9.0 Hz), 8.08 (m, 2H, 2 × CH_Ar_), 7.98 (d, 2H, 2 × CH_Ar_, J = 9.1 Hz), 7.83 (s, 1H, -CHCN_3_), 7.68 (m, 2H, 2 × CH_Ar_), 5.10 (m, 2H, *C*H_2_N), 4.91 (m, 2H, *C*H_2_NH), 4.25 (br. s, 2H, *C*H_carb_), 4.18 (br. s, 2H, *C*H_carb_), 3.47 (m, 4H, B*OC*H_2_, O*C*H_2_CH_2_C), 3.31 (m, 2H, CH_2_), 2.82 (m, 4H, 2 × CH_2_), 1.43 (m, 2H, CH_2_), 1.32 (m, 2H, CH_2_) ppm. ^11^*B* NMR (128 MHz, acetone-d_6_): 23.4 (1B, s), 4.3 (1*B*, d, J = 162 Hz), 0.4 (1*B*, d, J = 142), −2.6 (1*B*, d, J = 146 Hz), −4.6 (2*B*, d, J = 138 Hz), −7.4 (2*B*, d, J unsolved), −8.3 (4*B*, d, J = 134 Hz), −17.5 (2*B*, d, J = 164 Hz), −20.4 (2*B*, d, J = 148 Hz), −22.8 (1*B*, d, J unsolved), −28.6 (1*B*, d, J = 162 Hz) ppm. ^13^C NMR (101 MHz, acetone-d_6_): 158.7 (C_Ar_), 145.6 (C_Ar_), 139.9 (CN_3_CH), 135.8 (CH_Ar_), 125.3 (CH_Ar_), 124.4 (CH_Ar_), 123.2 (CN_3_CH), 119.0 (CH_Ar_), 112.9 (C_Ar_), 70.5 (OCH_2_), 69.1 (OCH_2_), 68.7 (OCH_2_), 53.9 (CH_carb_), 49.4 (NCH_2_), 48.7 (NCH_2_), 46.4 (CH_carb_), 31.6 (CH_2_), 29.4 (CH_2_), 26.3 (CH_2_), 22.7 (CH_2_) ppm. IR (KBr, н, cm*^−^*^1^): 2547 (BH), 1583 (triazole). Found: C 38.35, H 5.76, B 22.48, N 8.13; Calc. for C_28_H_49_B_18_CoN_5_O_2_Cs C 38.47, H 5.65, B 22.26, N 8.01. HRMS (ESI) m/z for [C_28_H_49_B_18_CoN_5_O_2_]^−^ calcd 741.5023 [M]^−^, found: 741.5028 [M]^−^.

### 3.2. Absorbance Spectroscopy

Absorption spectra were recorded on a Jasco v550 (Japan) spectrophotometer in a thermostatted cuvette with 1 cm optical path at 25 °C. A solution of 5 mkM compounds in 10 mM potassium phosphate buffer pH = 8.0 and a calf thymus DNA (Sigma-Aldrich) concentration in the range 0–80 µM (b.p.) was used to obtain spectral changes in DNA interactions.

### 3.3. Cells and MTT Assay

The human HCT116 colorectal carcinoma, MCF7 breast adenocarcinoma, A549 non-small cell lung carcinoma, and WI38 nonmalignant lung fibroblast cell lines were obtained from the European collection of authenticated cell cultures (ECACC; Salisbury, UK). All cells were grown in DMEM medium (Gibco™, Cork, Ireland) supplemented with 10% fetal bovine serum (Gibco™, Cork, Brazil). The cells were cultured in an incubator at 37 °C in a humidified 5% CO_2_ atmosphere and subcultured 2 times a week. The MTT (3-(4,5-dimethylthiazol-2-yl)-2,5-diphenyltetrazolium bromide) assay was performed as described earlier [46]. In brief, the cells were seeded in 96-well plates («*TPP*», Switzerland) at a 1 × 10^4^ cells/well in 100 µL. After 24 h incubation at 37 °C, the cells were incubated with the tested compounds in concentrations from 0 to 200 µM.

### 3.4. Crystallographic Data

At 100K, crystals of **2** (C_15_H_14_ClN_5_, M = 299.76) are orthorhombic, space group P2_1_2_1_2_1_: a = 7.0854(3), b = 11.0430(4), c = 17.4107(7) *E*, V = 1362.28(9) *E*^3^, Z = 4 (Z’ = 1), d_calc_ = 1.462 g·cm^−3^, F(000) = 624. Intensities of 14*,*766 reflections were measured with a Bruker D8 Quest diffractometer equipped with the Photon III detector [*λ*(MoK*α*) = 0.71072*E*, щ-scans, 2*θ* < 61*°*], and 3991 independent reflections [R_int_ = 0.0451] were used in further refinement. The structure was solved using the direct method and refined through the full-matrix least-squares technique against F^2^ in the anisotropic–isotropic approximation. The hydrogen atoms were found from the difference Fourier synthesis of electron density. All the hydrogen atoms were refined in the isotropic approximation without constraints imposed on the positional parameters. For **1**, the refinement converged to wR2 = 0.0844 and GOF = 0.959 for all independent reflections (R1 = 0.0364 was calculated against F for 3185 observed reflections with I > 2*σ*(I)). All calculations were performed using the SHELX [57] and OLEX2 [58] program packages. CCDC 2,257,438 contains the supplementary crystallographic data for **1**. These data can be obtained free of charge via https://www.ccdc.cam.ac.uk/structures/ (accessed on 3 July 2023) (or from the CCDC, 12 Union Road, Cambridge, CB21EZ, UK; or deposit@ccdc.cam.ac.uk). The X-ray diffraction study was performed using the equipment of the JRC PMR IGIC RAS.

The DFT calculations for the isolated cation of **1** as well for two conformations (relaxed and distorted) of the unsubstituted acridinium cation were performed using the Gaussian09 program [59]. The electronic energy calculations were performed using the def2TZVP basis set [60,61] and the PBE0 functional [62,63] with Grimme’s empirical dispersion correction [64] and Becke-Johnson damping [65]. The geometry optimization procedures were performed invoking the standard cutoff criteria and ultrafine grids. According to the normal mode calculations, both fully relaxed structures (the cation of **1** and the equilibrium unsubstituted acridinium cation) correspond to energy minima. The RDG and sign(л_2_)·c^®^ functions were calculated using the MultiWFN program [66], whereas the topological analysis of electron density function was performed in the AIMAll program [67].

## 4. Conclusions

The copper(I)-catalyzed 1,3-dipolar [3 + 2] cycloaddition reaction of cobalt bis(dicarbollide) alkynes derivatives with azidoacridine yield the corresponding products containing 18 atoms of boron per molecule. The novel conjugates demonstrated antiproliferative activity against two tumor and one non-tumor human cell lines. DNA interaction studies using absorbance spectroscopy showed the weak binding of the obtained compounds with DNA. Among all other compounds, the acridine conjugate obtained from the 1,4-dioxane derivative of cobalt bis(dicarbollide) and propargyl alcohol showed the best result (the largest spectral changes were observed for this compound). It should be noted that the preliminary results indicated some potential, suggesting binding to DNA. Thus, the toxic boronated acridines presented in this work contain novel, unexplored structural features whose relevance in the field deserves investigation in order to achieve still-better non-toxic BNCT agents, capable of binding to DNA. Further efforts are warranted to explore the effect of other kinds of boronated substituents on the 9-aminoacridine scaffold on the activity and toxicity of boronated acridines. As compounds with a possibly more affinitive interaction with DNA, the introduction of active groups carrying a positive charge could be suggested, which would increase water solubility and make the acridine core more affinitive to DNA, possibly enhancing binding to DNA as a target.

## Figures and Tables

**Table 1:** Antiproliferative activity of conjugates **7**–**10** and cisplatin against human cancer cells.

Cell Line	IC_50_, µM				
	7	8	9	10	Cisplatin [55]
WI38	21 ± 4	21 ± 5	33 ± 3	34 ± 8	8 ± 3
A549	>200	>200	>200	>200	13 ± 3
HCT116	49 ± 20	61 ± 25	43 ± 18	41 ± 1	13 ± 4
MCF7	15 ± 3	25 ± 6	28 ± 3	13 ± 3	30 ± 9

## Data Availability

The data presented in this study are available in the Supplementary Materials.

## Supplementary Materials

The following supporting information can be downloaded at: https://www.mdpi.com/1420-3049/28/18/6636/s1, Figure S1 with main crystallographic data for **2.** Figure S2 with the area of substituent in the isolated optimized cation from **2**. Figure S3 with changes of absorbance spectra upon DNA interaction for conjugates **7,9,10**. Figures S4–S8: ESI-HRMS spectra of compounds **2**, **7**–**10**, Figures S9–S13: IR spectra of compounds **2**, **7**–**10**, Figures S14–S31: ^1^H, ^11^B{^1^H}, ^11^B and ^13^C{^1^H} spectra of compounds **2**, **7**–**10**.

## Sample Availability

Samples of compounds **7**–**10** are available from the authors.
