# Supplementary material for: New Boron Containing Acridines: Synthesis and Preliminary Biological Study
Source: Molecules. 2023 Sep 15;28(18):6636. doi: 10.3390/molecules28186636 (PMC10650824; doi:10.3390/molecules28186636)

# checkCIF/PLATON report

Structure factors have been supplied for datablock(s) a

THIS REPORT IS FOR GUIDANCE ONLY. IF USED AS PART OF A REVIEW PROCEDURE FOR PUBLICATION, IT SHOULD NOT REPLACE THE EXPERTISE OF AN EXPERIENCED CRYSTALLOGRAPHIC REFEREE.

No syntax errors found. CIF dictionary Interpreting this report

# Datablock: a

---

```
Bond precision:   C-C = 0.0031 A          Wavelength=0.71073

Cell:               a=7.0854(3)      b=11.0430(4)      c=17.4107(7)
                    alpha=90         beta=90           gamma=90
Temperature:        140 K

                  Calculated                   Reported
Volume            1362.28(9)                   1362.28(9)
Space group       P 21 21 21                   P 21 21 21
Hall group        P 2ac 2ab                    P 2ac 2ab
Moiety formula    C15 H14 N5, Cl               C15 H14 N5, Cl
Sum formula       C15 H14 Cl N5                C15 H14 Cl N5
Mr                299.76                       299.76
Dx,g cm-3         1.462                        1.462
Z                 4                            4
Mu (mm-1)         0.281                        0.281
F000              624.0                        624.0
F000'             624.76
h,k,lmax          9,15,24                      9,15,24
Nref              4001[ 2294]                  3991
Tmin,Tmax         0.953,0.994                  0.921,0.995
Tmin'             0.942

Correction method= # Reported T Limits: Tmin=0.921 Tmax=0.995
AbsCorr = MULTI-SCAN

Data completeness= 1.74/1.00       Theta(max)= 30.050

R(reflections)= 0.0364( 3185)                         wR2(reflections)=
                                                      0.0844( 3991)
S = 0.959                  Npar= 246
```

The following ALERTS were generated. Each ALERT has the format
**test-name_ALERT_alert-type_alert-level.**
Click on the hyperlinks for more details of the test.

## Alert level C

```
PLAT222_ALERT_3_C NonSolvent Resd 1  H   Uiso(max)/Uiso(min) Range        6.4 Ratio
PLAT230_ALERT_2_C Hirshfeld Test Diff for    N3       --N4       .        5.1 s.u.
PLAT245_ALERT_2_C U(iso) H4        Smaller than U(eq) C4        by      0.017 Ang**2
PLAT245_ALERT_2_C U(iso) H10       Smaller than U(eq) C10       by      0.018 Ang**2
```

## Alert level G

```
PLAT912_ALERT_4_G Missing # of FCF Reflections Above STh/L=  0.600          1 Note
PLAT978_ALERT_2_G Number C-C Bonds with Positive Residual Density.          9 Info
```

0 **ALERT level A** = Most likely a serious problem - resolve or explain
0 **ALERT level B** = A potentially serious problem, consider carefully
4 **ALERT level C** = Check. Ensure it is not caused by an omission or oversight
2 **ALERT level G** = General information/check it is not something unexpected

0 ALERT type 1 CIF construction/syntax error, inconsistent or missing data
4 ALERT type 2 Indicator that the structure model may be wrong or deficient
1 ALERT type 3 Indicator that the structure quality may be low
1 ALERT type 4 Improvement, methodology, query or suggestion
0 ALERT type 5 Informative message, check

It is advisable to attempt to resolve as many as possible of the alerts in all categories. Often the minor alerts point to easily fixed oversights, errors and omissions in your CIF or refinement strategy, so attention to these fine details can be worthwhile. In order to resolve some of the more serious problems it may be necessary to carry out additional measurements or structure refinements. However, the purpose of your study may justify the reported deviations and the more serious of these should normally be commented upon in the discussion or experimental section of a paper or in the "special_details" fields of the CIF. checkCIF was carefully designed to identify outliers and unusual parameters, but every test has its limitations and alerts that are not important in a particular case may appear. Conversely, the absence of alerts does not guarantee there are no aspects of the results needing attention. It is up to the individual to critically assess their own results and, if necessary, seek expert advice.

**Publication of your CIF in IUCr journals**

A basic structural check has been run on your CIF. These basic checks will be run on all CIFs submitted for publication in IUCr journals (*Acta Crystallographica*, *Journal of Applied Crystallography*, *Journal of Synchrotron Radiation*); however, if you intend to submit to *Acta Crystallographica Section C* or *E* or *IUCrData*, you should make sure that full publication checks are run on the final version of your CIF prior to submission.

**Publication of your CIF in other journals**

Please refer to the *Notes for Authors* of the relevant journal for any special instructions relating to CIF submission.

---

**PLATON version of 28/11/2022; check.def file version of 28/11/2022**

**Datablock a** - ellipsoid plot

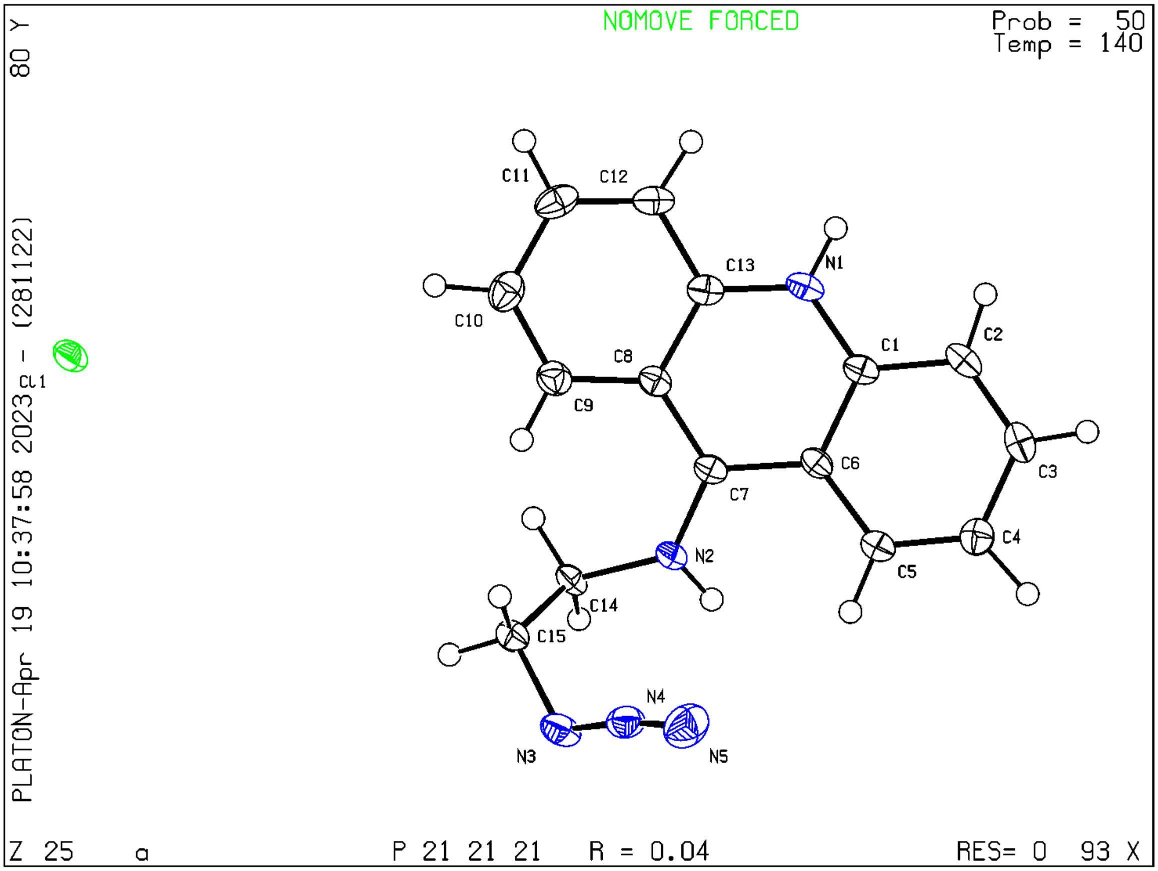

Supplement: Supplementary file 1 [file molecules-28-06636-s001.zip › checkcif_compound 2.pdf]
