# Supplementary material for: New Boron Containing Acridines: Synthesis and Preliminary Biological Study
Source: Molecules. 2023 Sep 15;28(18):6636. doi: 10.3390/molecules28186636 (PMC10650824; doi:10.3390/molecules28186636)
Supplement: Supplementary file 1 [file molecules-28-06636-s001.zip › Supporting Information.pdf]

SUPPORTING INFORMATION

New Boron Containing Acridines: Synthesis and Preliminary Biological Study

Anna A. Druzina[1,*], Nadezhda V. Dudarova[1], Ivan. V. Ananyev[2], Anastasia A. Antonets[3], Dmitry N. Kaluzhny[4], Alexey A. Nazarov[3], Igor B. Sivaev[1,5] and Vladimir I. Bregadze[1,†]

1 A.N. Nesmeyanov Institute of Organoelement Compounds, Russian Academy of Sciences, 28 Vavilov Str., 119991 Moscow, Russia; ilinova_anna@mail.ru (A.A.D.); nadezjdino_96@mail.ru (N.V.D.); sivaev@ineos.ac.ru (I.B.S.); bre@ineos.ac.ru (V.I.B).

2 N.S. Kurnakov Institute of General and Inorganic Chemistry, Russian Academy of Sciences, 31 Leninskii pr., 119991 Moscow, Russia; i.ananyev@gmail.com (I.V.A.).

3 Department of Chemistry, M.V. Lomonosov Moscow State University, 1/3 Leninskie Gory, 119991 Moscow, Russia; antonets.anastasia.a@gmail.com (A.A.A.); nazarov@med.chem.msu.ru (A.A.N.).

4 V.A. Engelhardt Institute of Molecular Biology, Russian Academy of Sciences, 32 Vavilov Str., 11991 Moscow, Russia; uzhny@mail.ru (D.N.K.).

5 Basic Department of Chemistry of Innovative Materials and Technologies, G.V. Plekhanov Russian University of Economics, 36 Stremyannyi Line, Moscow 117997, Russia; sivaev@ineos.ac.ru (I.B.S.).

* Correspondence: ilinova_anna@mail.ru; Tel.: +7-926-404-5566 (A.A.D.)

† Dedicated to John Kennedy on the occasion of his 80th Jubilee and in recognition of his outstanding contributions to inorganic and organometallic chemistry.

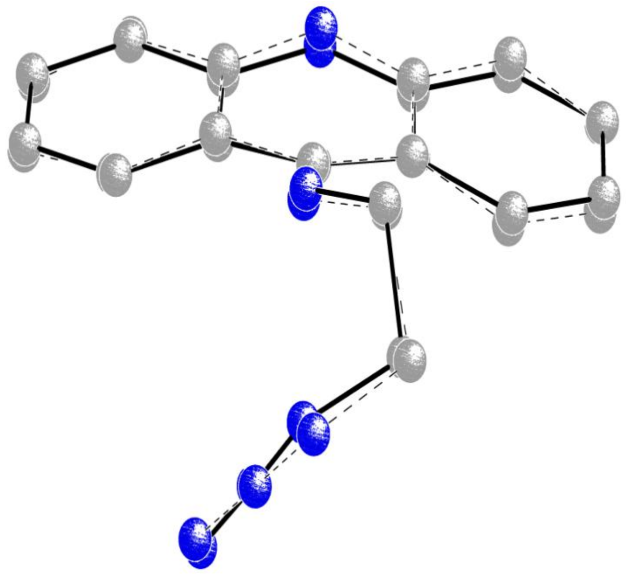

Figure S1. The best root-mean-square overlap for the isolated optimized (full lines) and crystal (dashed lines) structure of the substituted acridinium cation from **2**.

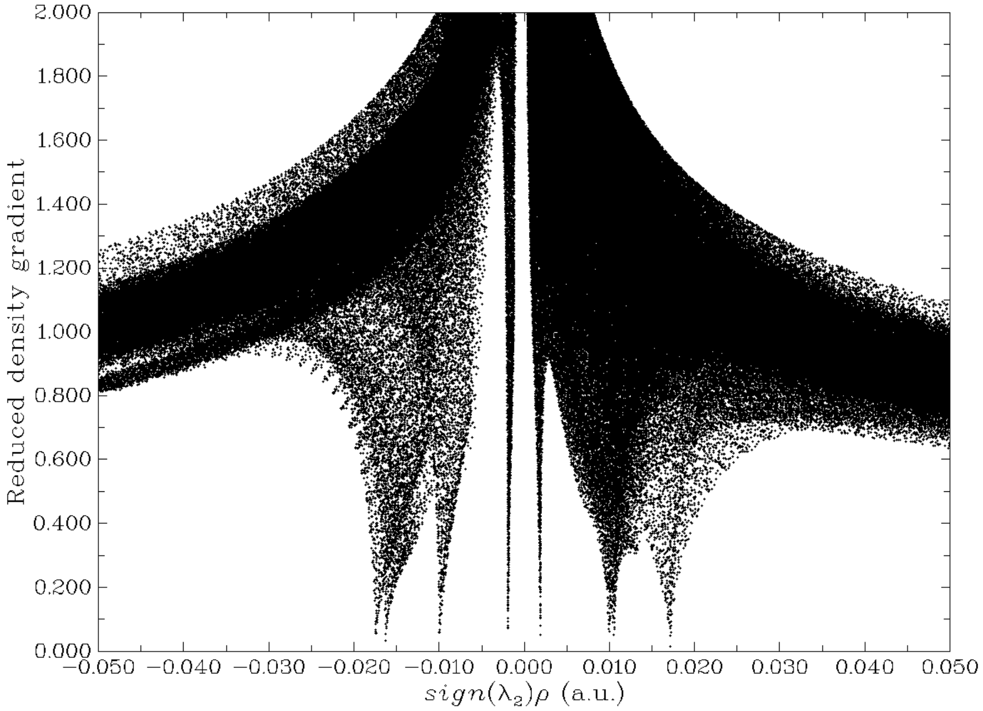

Figure S2. The RDG/sign(λ_2)·ϱ(**r**) in the area of substituent in the isolated optimized cation from **2**.

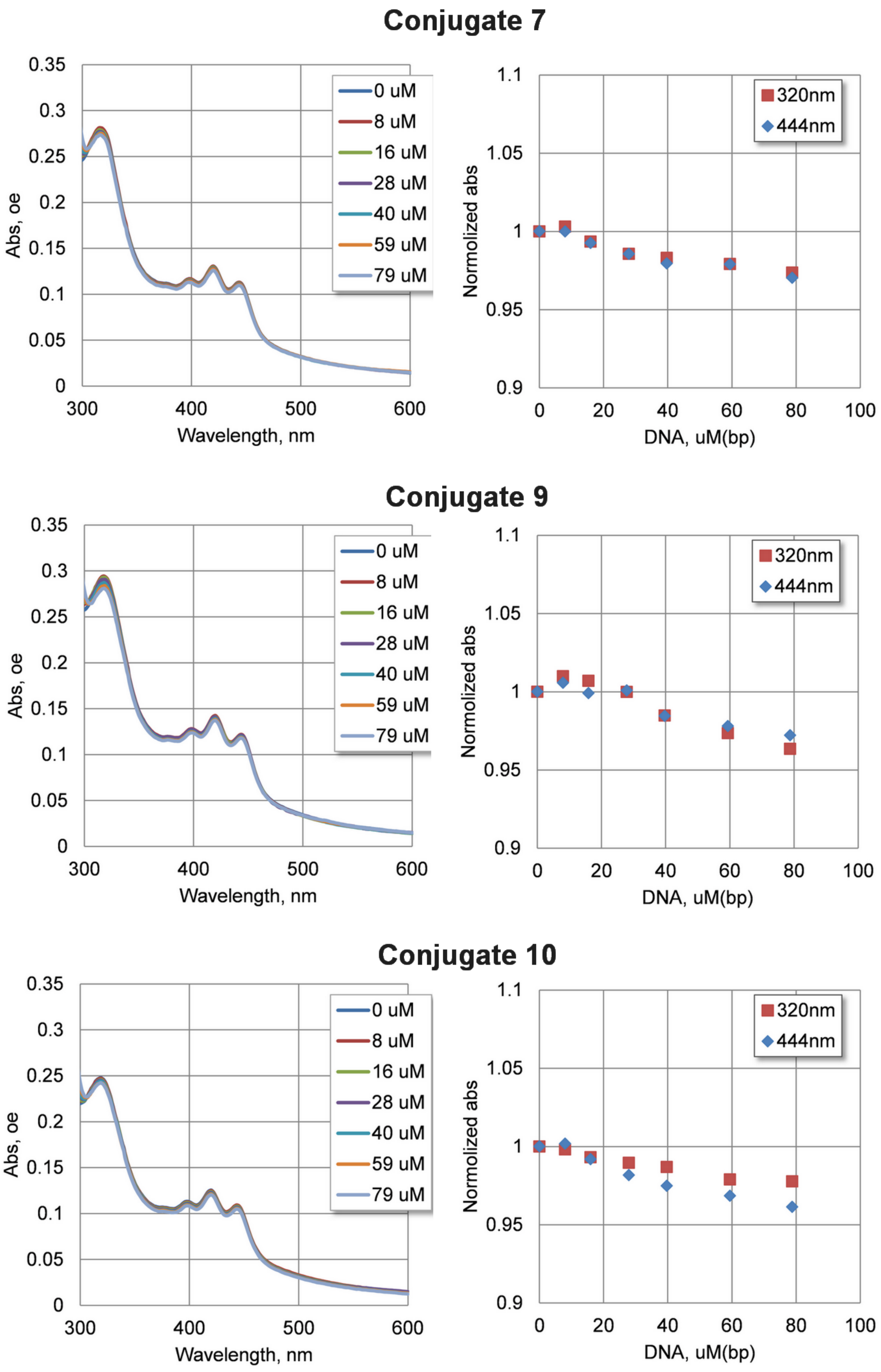

Figure S3. Changes of absorbance spectra upon DNA interaction for conjugates **7,9,10**. 5 mkM compounds in 10 mM potassium phosphate buffer pH=8.0 and a calf thymus DNA in the range 0-80 μM (b.p.).

^{1}H, $^{11}B\{^{1}H\}$, ^{11}B and $^{13}C\{^{1}H\}$ NMR, IR and high-resolution mass spectra of compounds **2**, **7-10**

Display Report

Analysis Info

Analysis Name	D:\Data\Chizhov\INEOS\Bregadze\Druzina\Apr_17_2023\da-059_&clblow.d
Method	tune_low.m
Sample Name	/CHIZ DA-059
Comment	CH3CN 100 %, dil. 200, calibrant added
Acquisition Date	17.04.2023 16:43:51
Operator	BDAL@DE
Instrument / Ser#	micrOTOF 10248

Acquisition Parameter

Source Type	ESI	Ion Polarity	Positive	Set Nebulizer	0.4 Bar
Focus	Not active			Set Dry Heater	180 °C
Scan Begin	50 m/z	Set Capillary	4500 V	Set Dry Gas	4.0 l/min
Scan End	3000 m/z	Set End Plate Offset	-500 V	Set Divert Valve	Waste

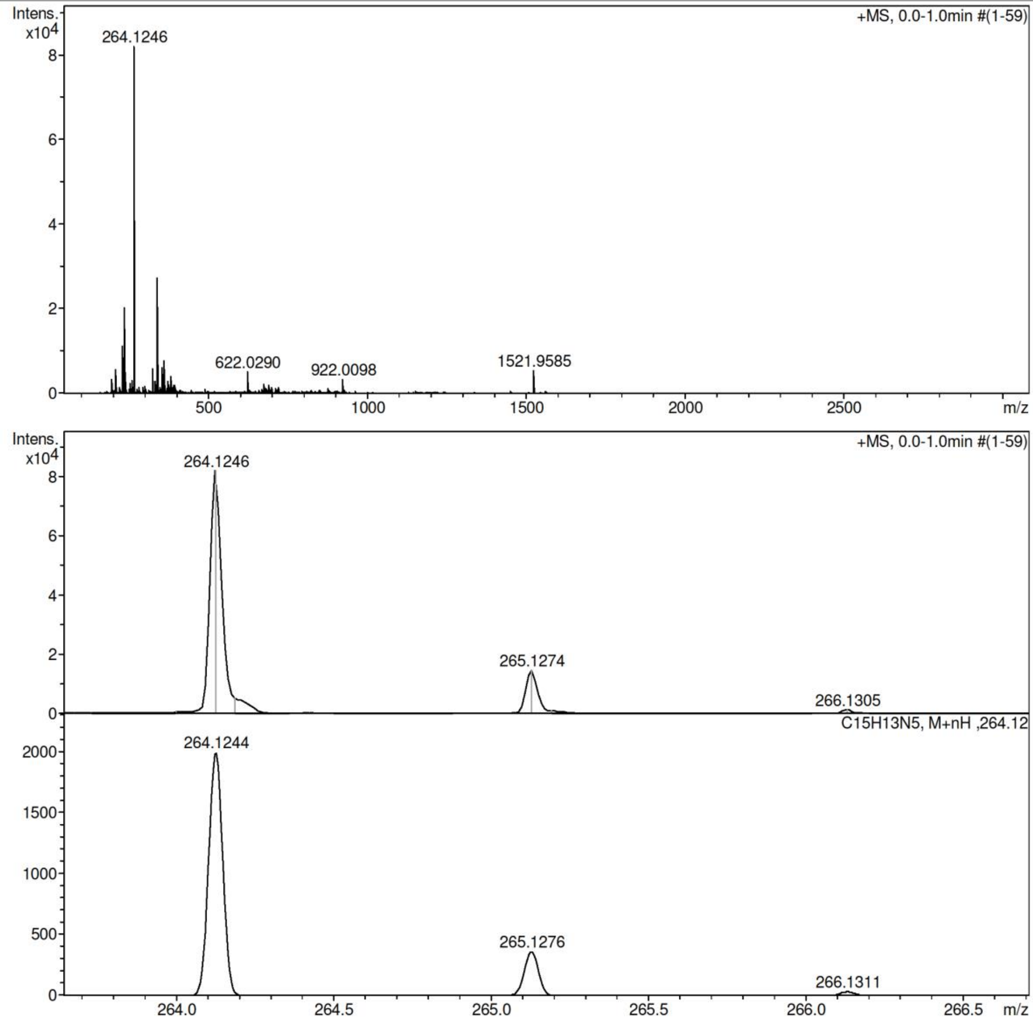

Bruker Compass DataAnalysis 4.0   printed: 17.04.2023 17:30:38   Page 1 of 1

Figure S4. ESI-HRMS spectrum of compound **2**

Display Report

Analysis Info

Analysis Name D:\Data\Chizhov\INEOS\Druzina\Apr_18_2023\da-051_&clb-.d
Method tune_wide_neg.m
Sample Name /CHIZ DA-051
Comment CH3CN 100 %, dil. 200, calibrant added

Acquisition Date 18.04.2023 17:40:18
Operator BDAL@DE
Instrument / Ser# maXis 43

Acquisition Parameter

Source Type	ESI	Ion Polarity	Negative	Set Nebulizer	0.4 Bar
Focus	Active			Set Dry Heater	180 °C
Scan Begin	50 m/z	Set Capillary	3000 V	Set Dry Gas	4.0 l/min
Scan End	3000 m/z	Set End Plate Offset	-500 V	Set Divert Valve	Waste

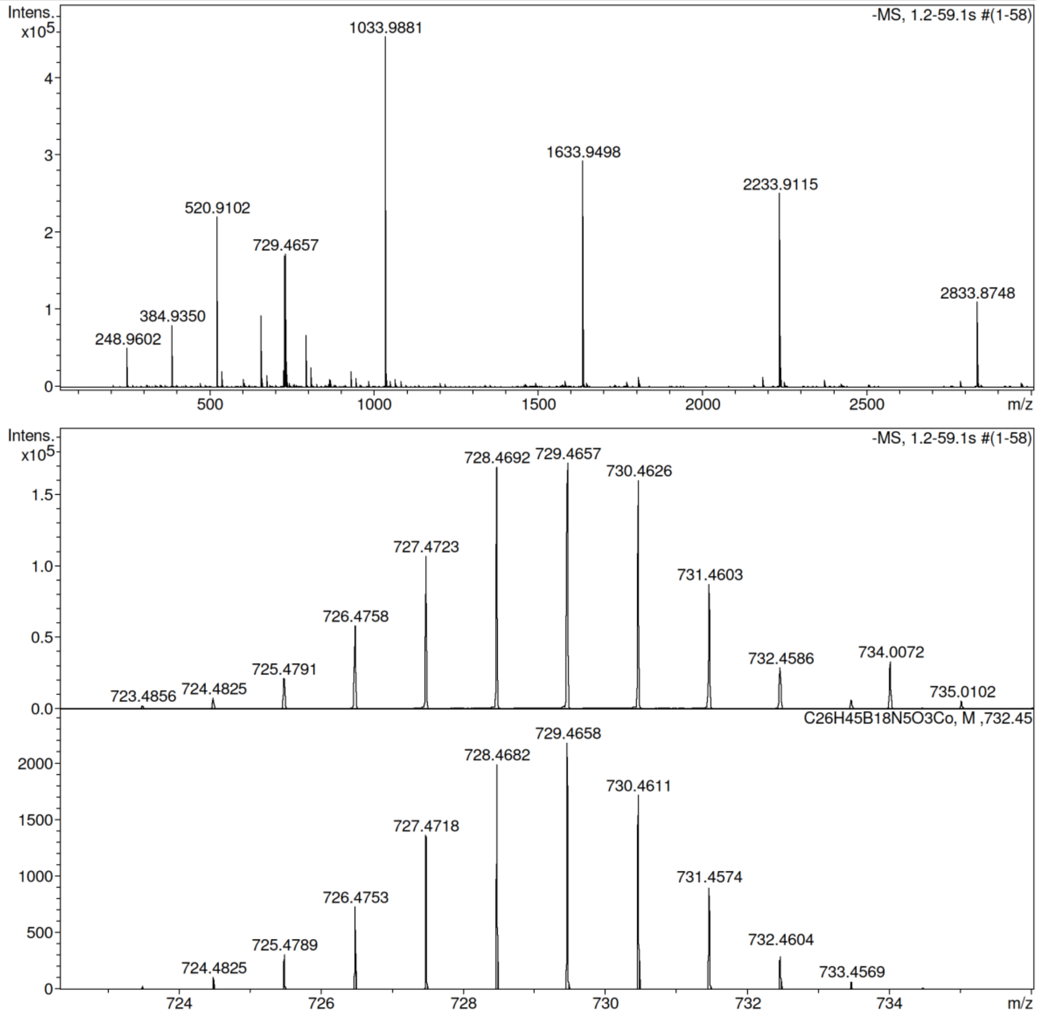

Bruker Compass DataAnalysis 4.0 printed: 18.04.2023 18:39:06 Page 1 of 1

Figure S5. ESI-HRMS spectrum of compound **7**

Display Report

Analysis Info

Analysis Name	D:\Data\Chizhov\INEOS\Druzina\Apr_18_2023\da-052_&clb-.d	Acquisition Date	18.04.2023 17:47:37
Method	tune_wide_neg.m	Operator	BDAL@DE
Sample Name	/CHIZ DA-052	Instrument / Ser#	maXis 43
Comment	CH3CN 100 %, dil. 200, calibrant added		

Acquisition Parameter

Source Type	ESI	Ion Polarity	Negative	Set Nebulizer	0.4 Bar
Focus	Active			Set Dry Heater	180 °C
Scan Begin	50 m/z	Set Capillary	3000 V	Set Dry Gas	4.0 l/min
Scan End	3000 m/z	Set End Plate Offset	-500 V	Set Divert Valve	Waste

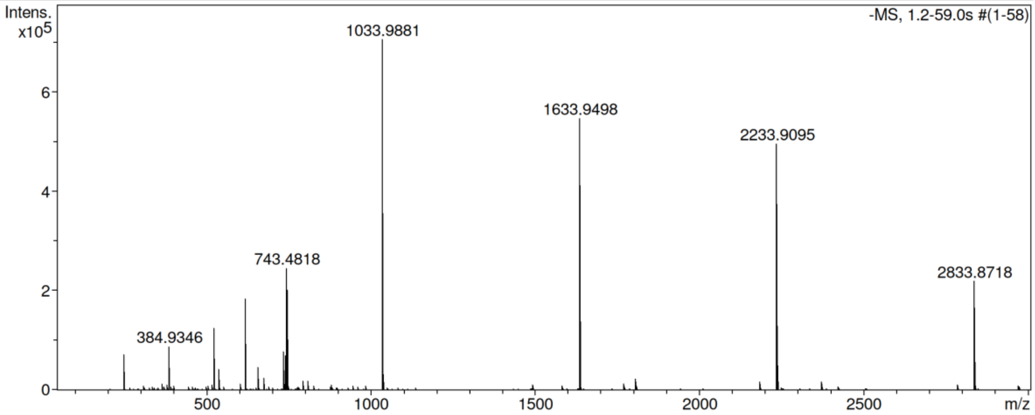

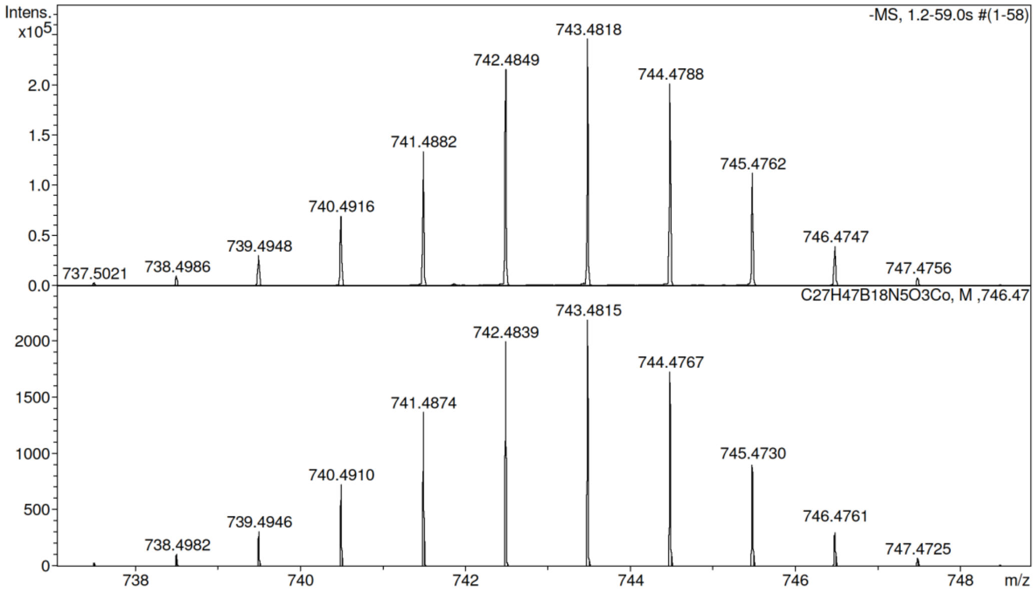

Bruker Compass DataAnalysis 4.0 printed: 18.04.2023 18:43:40 Page 1 of 1

Figure S6. ESI-HRMS spectrum of compound **8**

Display Report

Analysis Info

Analysis Name	D:\Data\Chizhov\INEOS\Druzina\Apr_18_2023\da-053_&clb-.d
Method	tune_wide_neg.m
Sample Name	/CHIZ DA-053
Comment	CH3CN 100 %, dil. 200, calibrant added
Acquisition Date	18.04.2023 17:53:03
Operator	BDAL@DE
Instrument / Ser#	maXis 43

Acquisition Parameter

Source Type	ESI	Ion Polarity	Negative	Set Nebulizer	0.4 Bar
Focus	Active			Set Dry Heater	180 °C
Scan Begin	50 m/z	Set Capillary	3000 V	Set Dry Gas	4.0 l/min
Scan End	3000 m/z	Set End Plate Offset	-500 V	Set Divert Valve	Waste

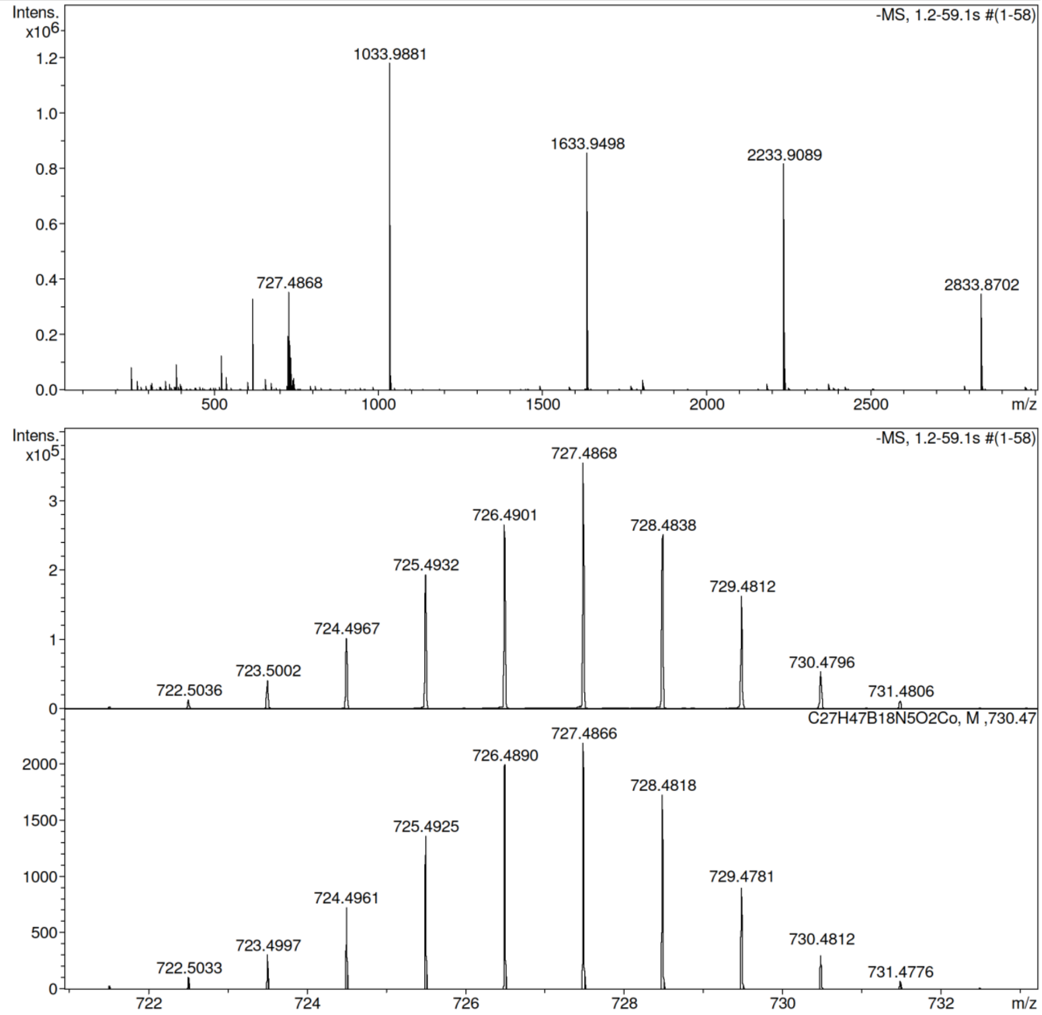

Figure S7. ESI-HRMS spectrum of compound **9**

Display Report

Analysis Info

Analysis Name	D:\Data\Chizhov\INEOS\Druzina\Apr_18_2023\da-054_&clb-.d	Acquisition Date	18.04.2023 17:59:10
Method	tune_wide_neg.m	Operator	BDAL@DE
Sample Name	/CHIZ DA-054	Instrument / Ser#	maXis 43
Comment	CH3CN 100 %, dil. 200, calibrant added		

Acquisition Parameter

Source Type	ESI	Ion Polarity	Negative	Set Nebulizer	0.4 Bar
Focus	Active			Set Dry Heater	180 °C
Scan Begin	50 m/z	Set Capillary	3000 V	Set Dry Gas	4.0 l/min
Scan End	3000 m/z	Set End Plate Offset	-500 V	Set Divert Valve	Waste

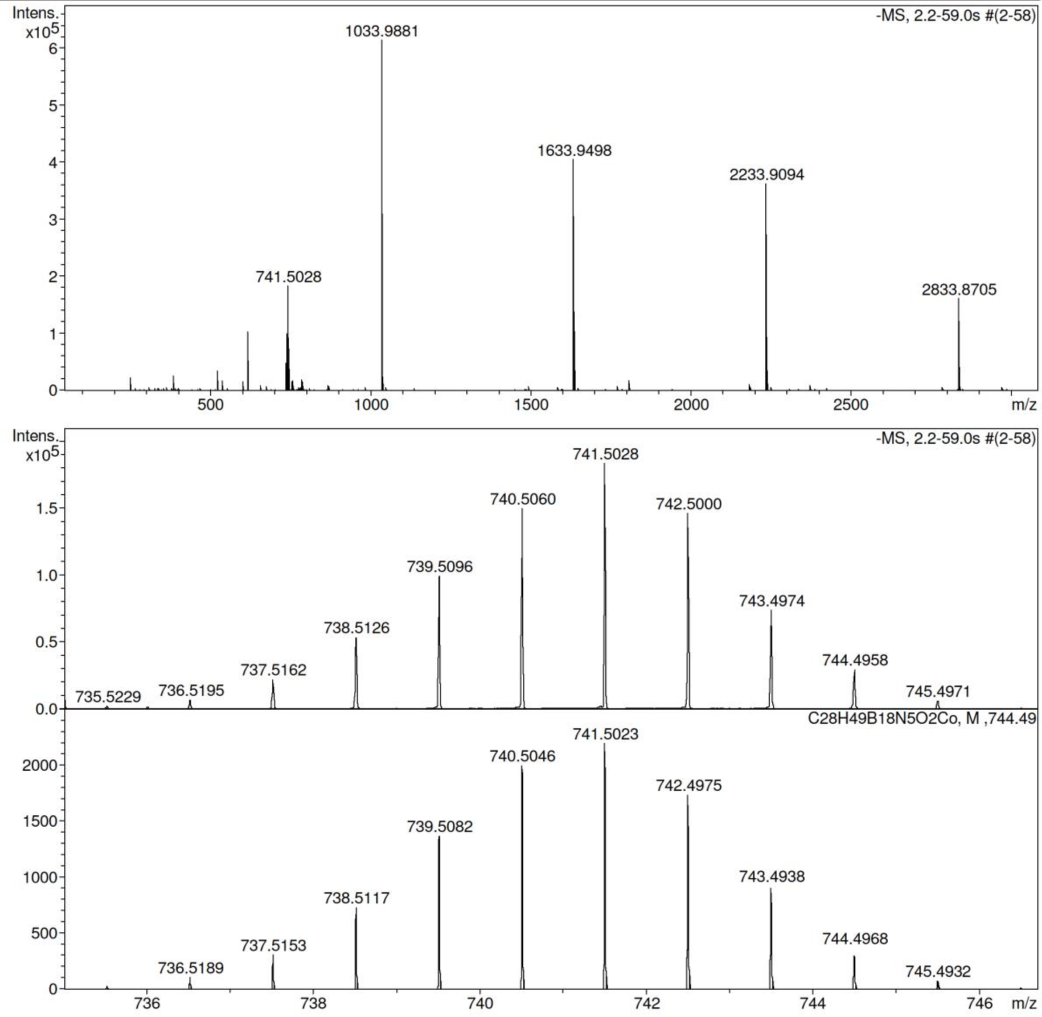

Figure S8. ESI-HRMS spectrum of compound **10**

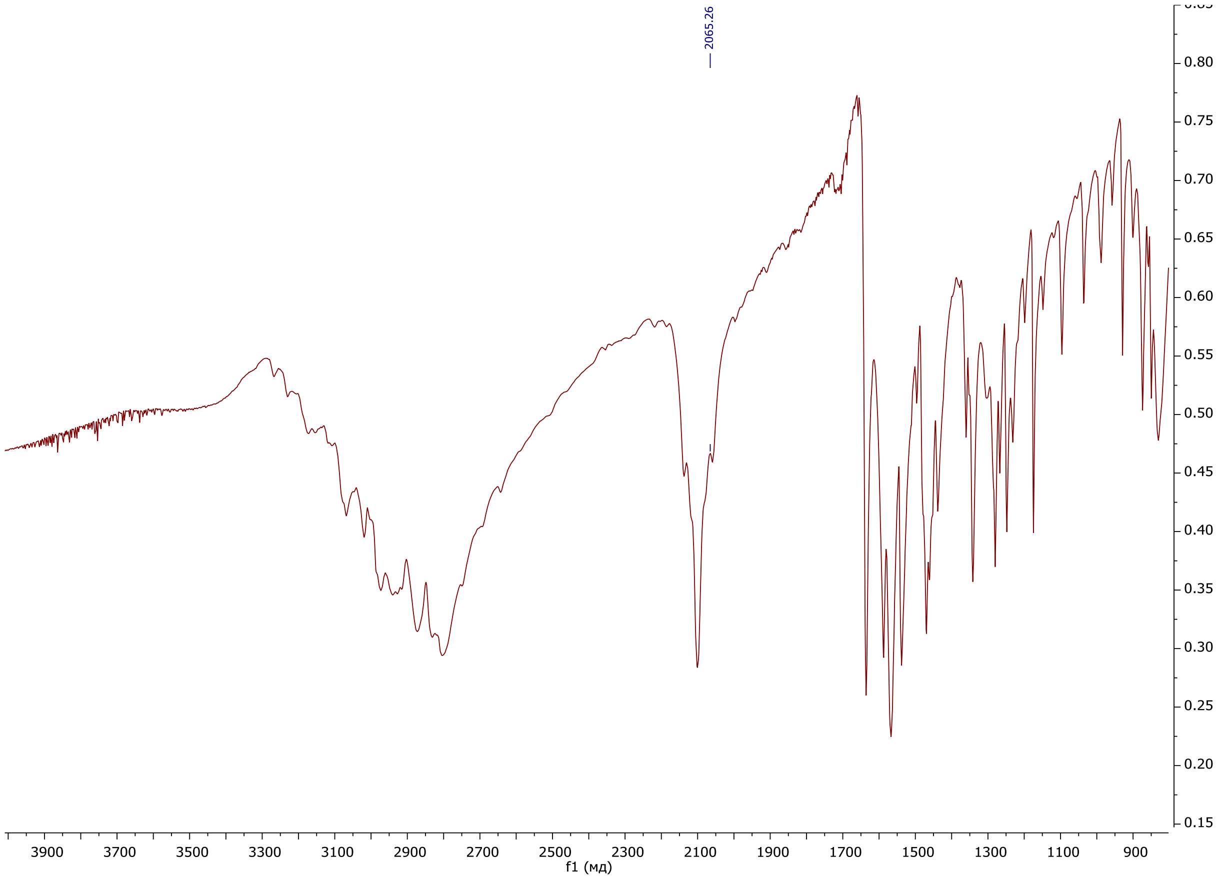

Figure S9. IR spectrum of compound **2**

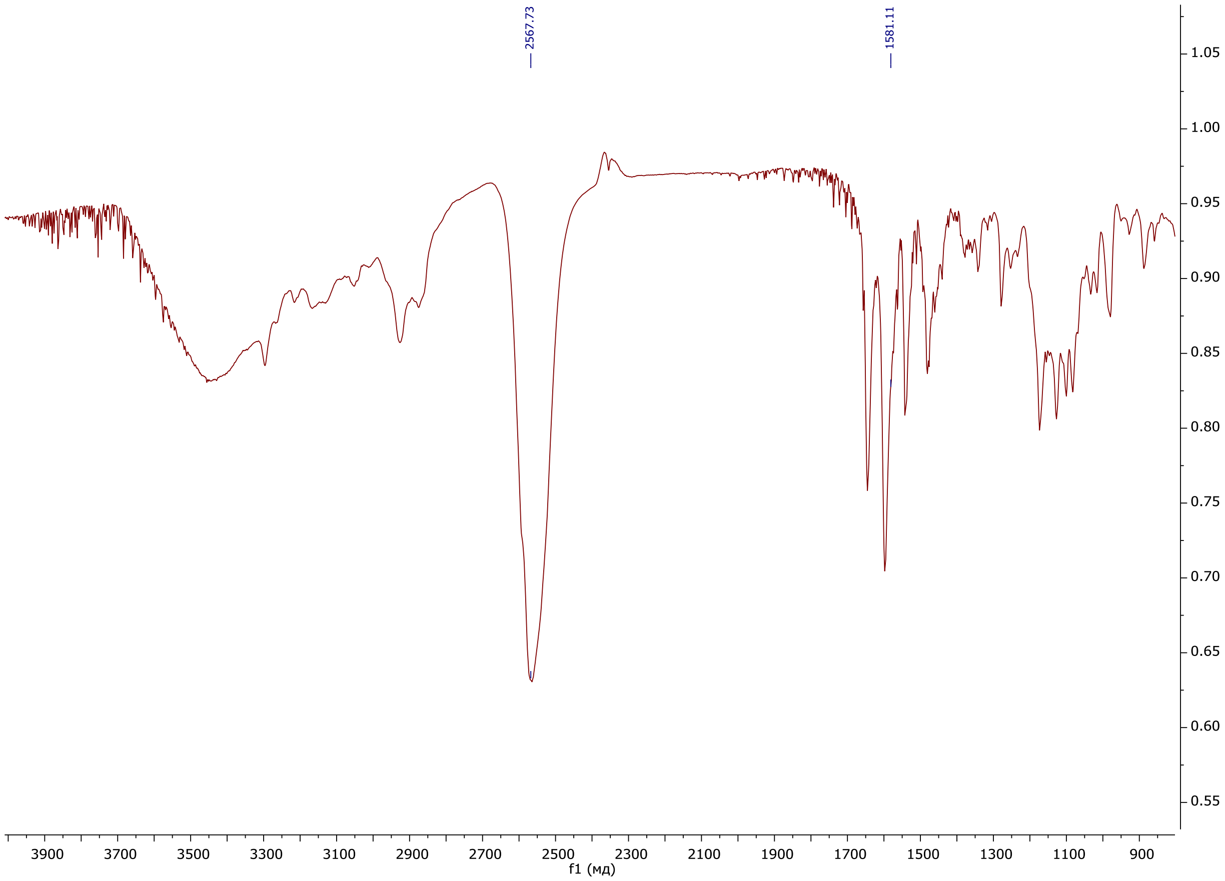

Figure S10. IR spectrum of compound **7**

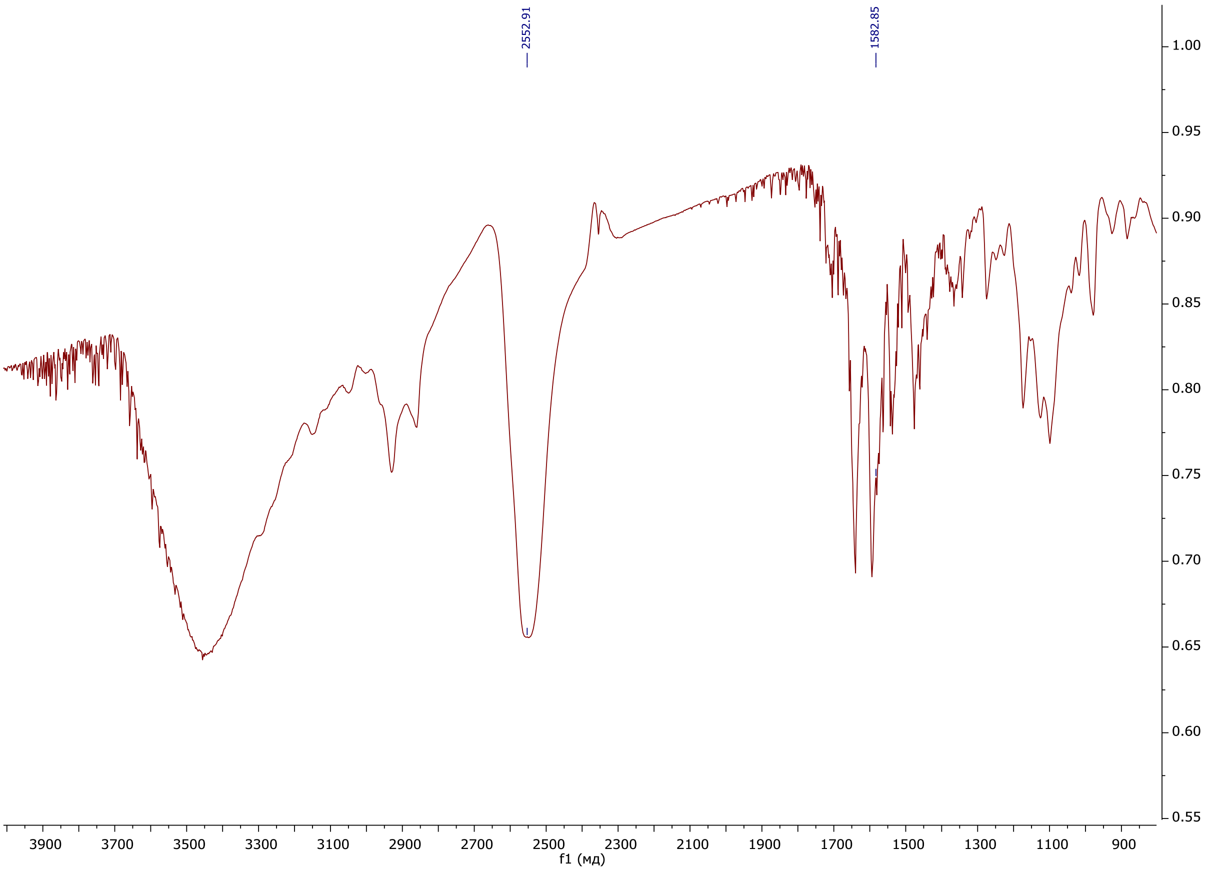

Figure S11. IR spectrum of compound **8**

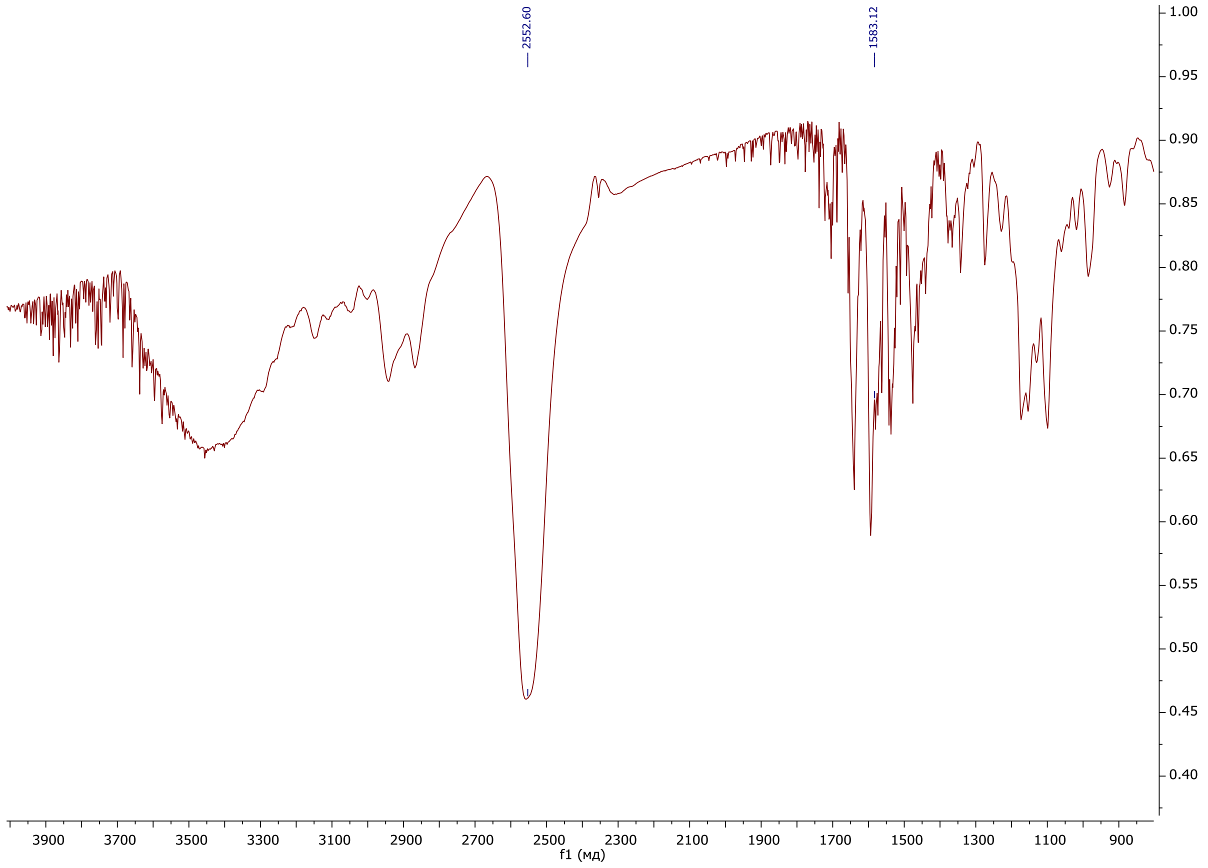

Figure S12. IR spectrum of compound **9**

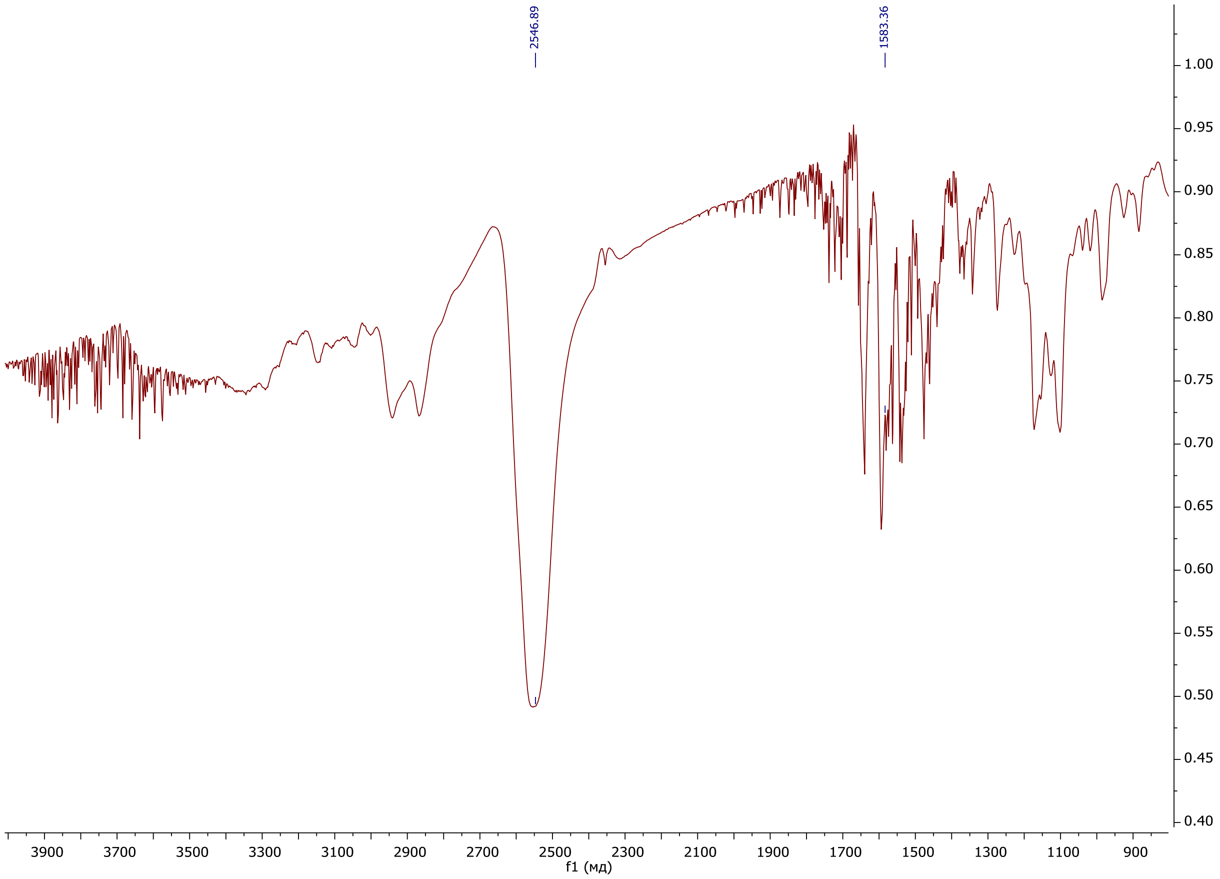

Figure S13. IR spectrum of compound **10**

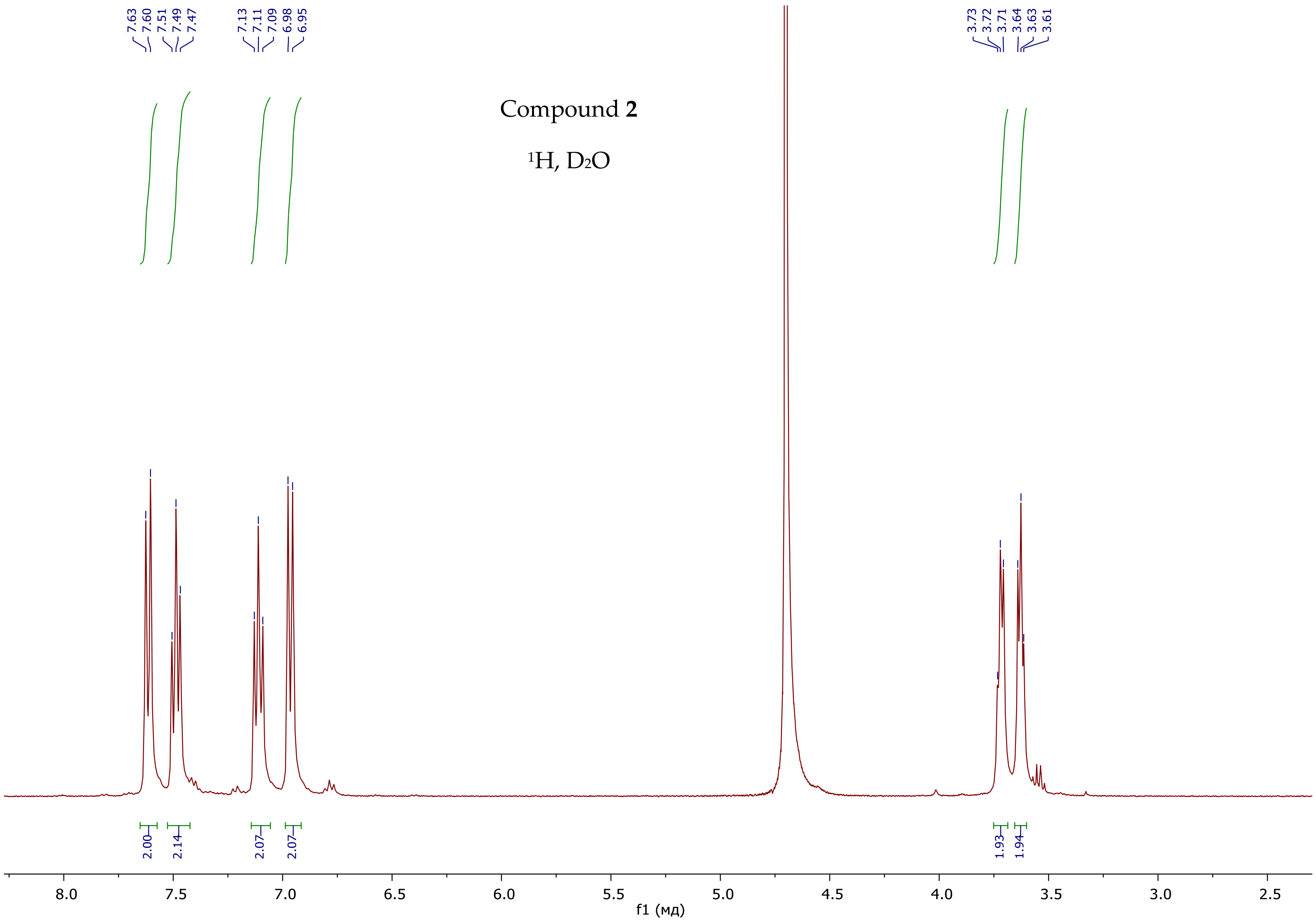

Figure S14. ^{1}H NMR spectrum of compound **2**

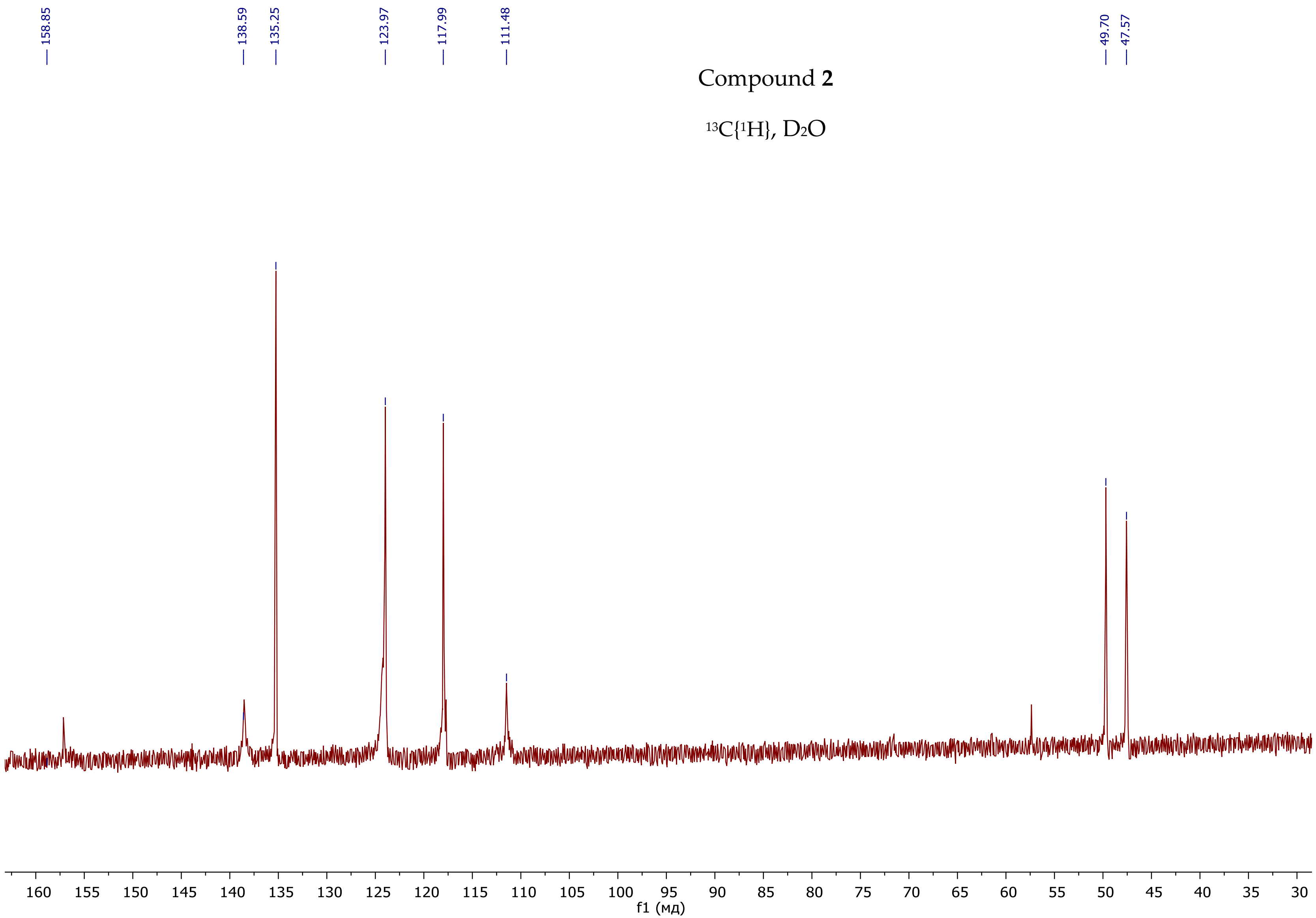

Figure S15. $^{13}C\{^1H\}$ NMR spectrum of compound **2**

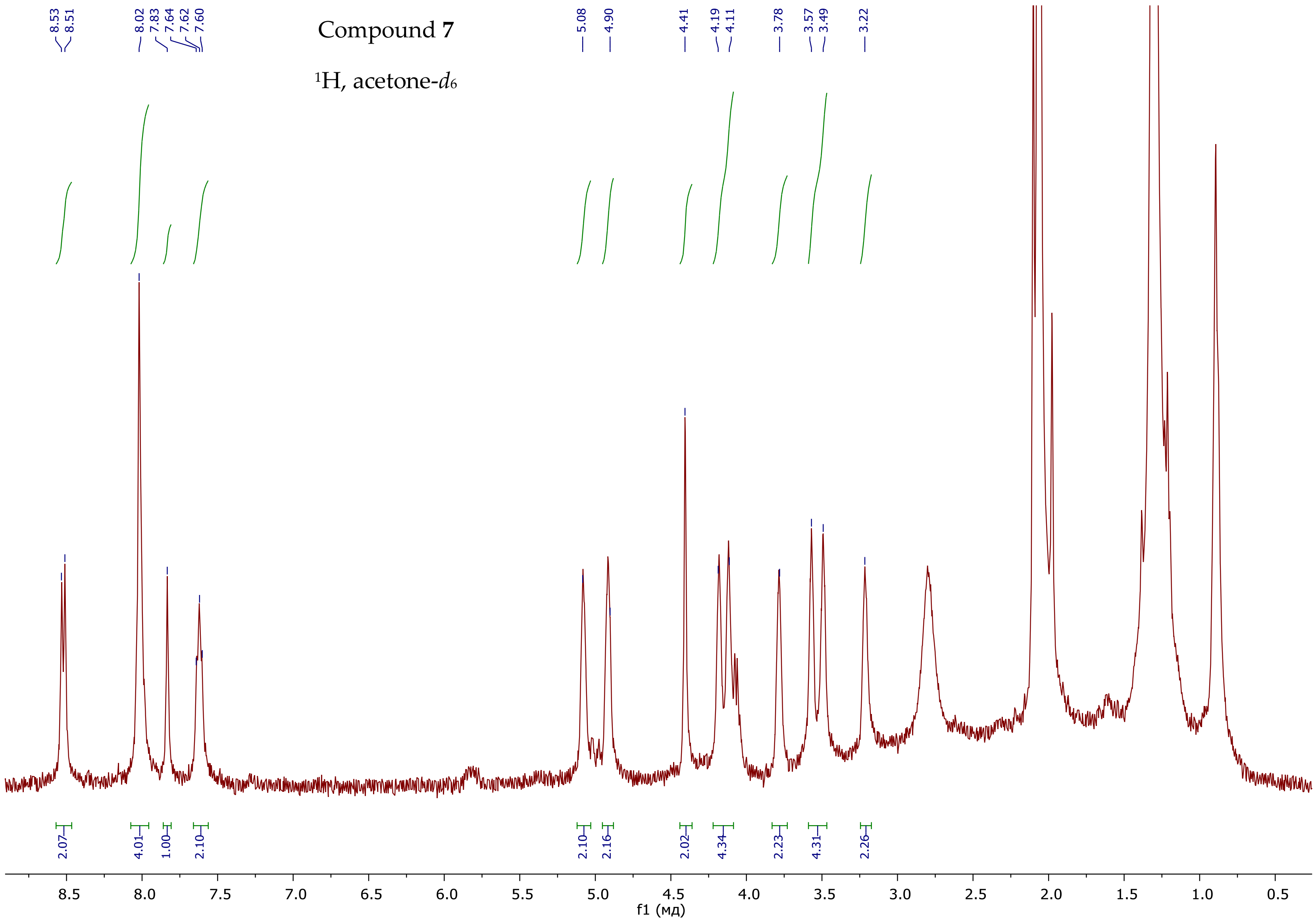

Figure S16. ^{1}H NMR spectrum of compound **7**

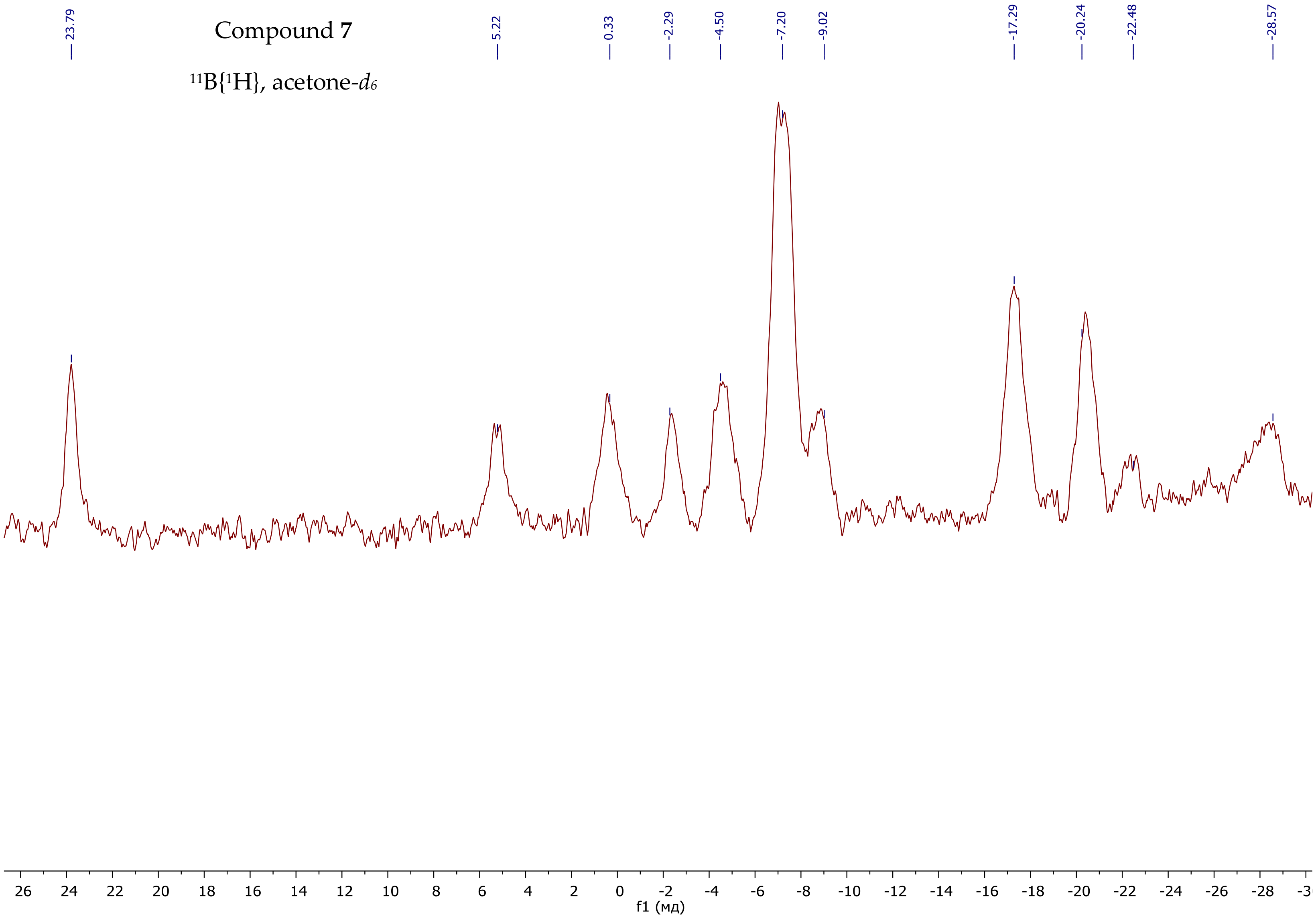

Figure S17. $^{11}B\{^1H\}$ NMR spectrum of compound **7**

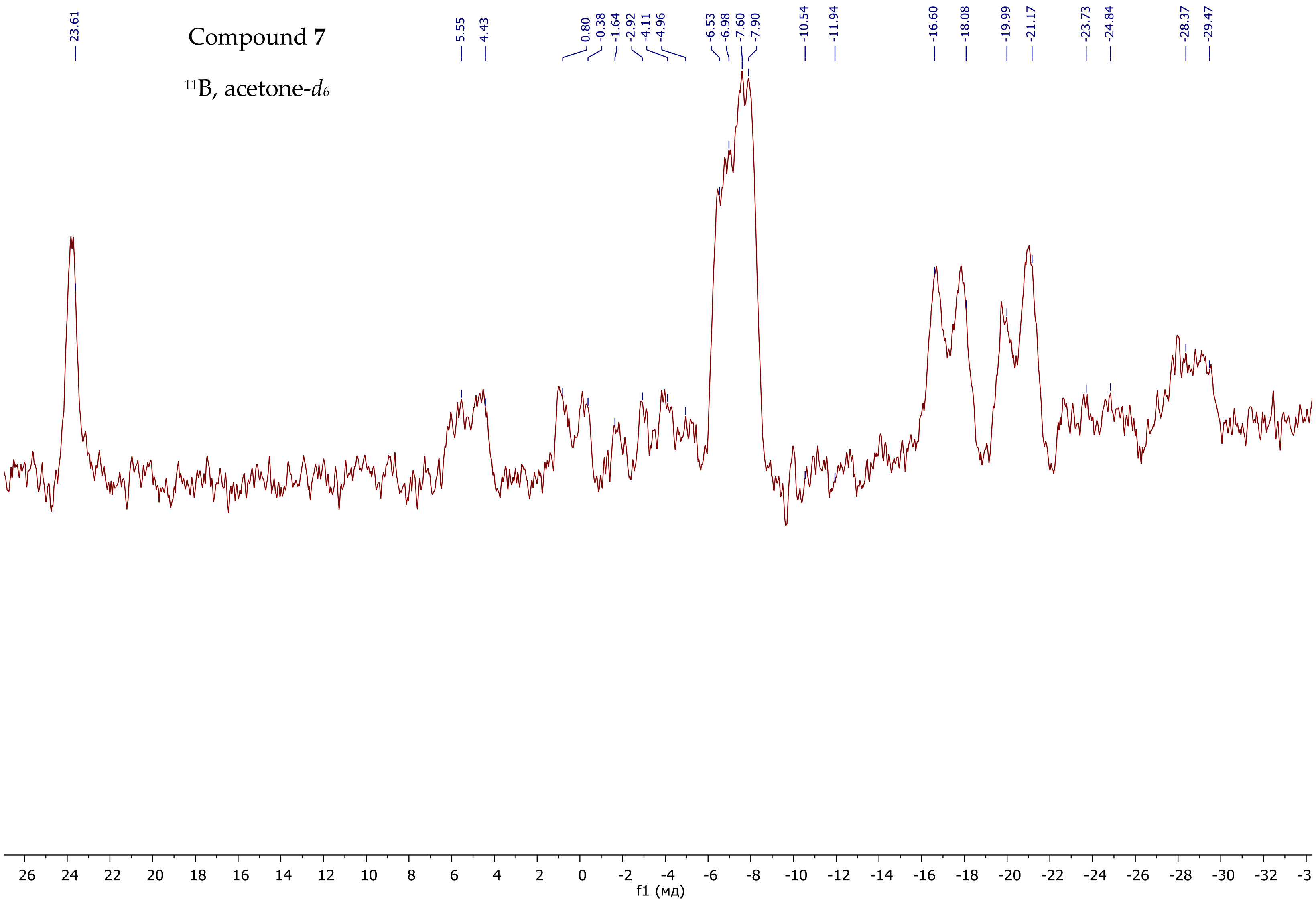

Figure S18. ^{11}B NMR spectrum of compound **7**

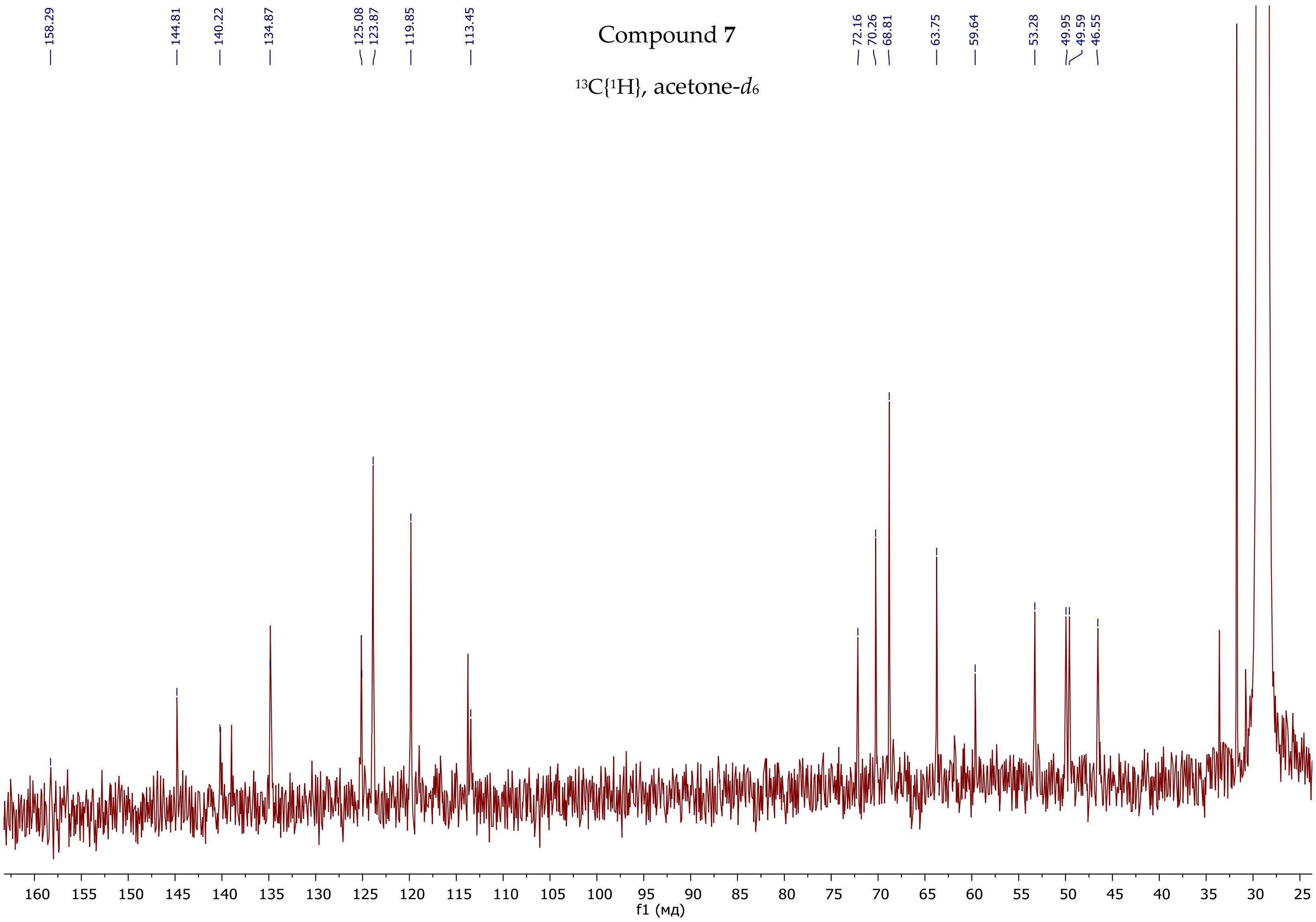

Figure S19. $^{13}C\{^1H\}$ NMR spectrum of compound **7**

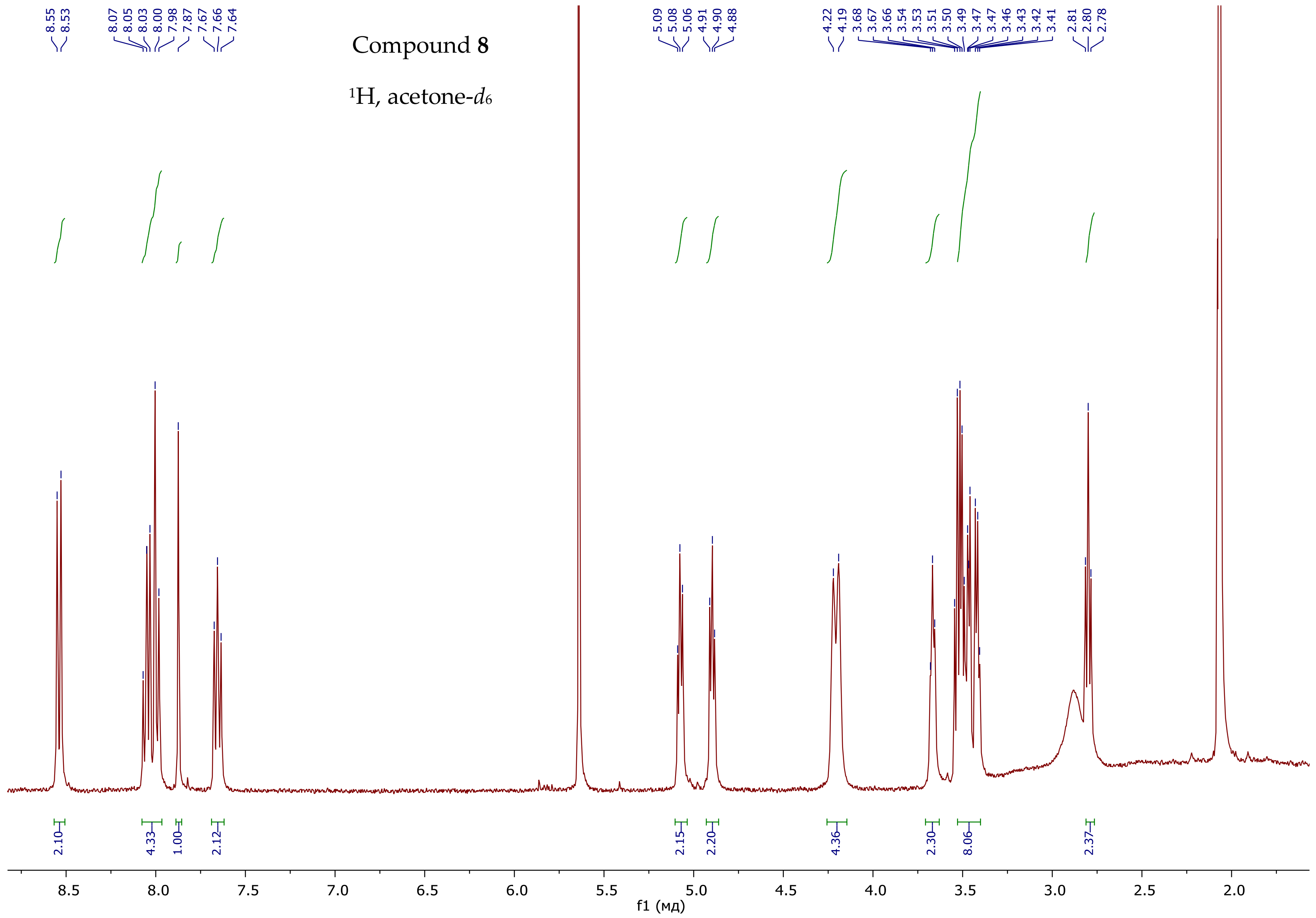

Figure S20. ^{1}H NMR spectrum of compound **8**

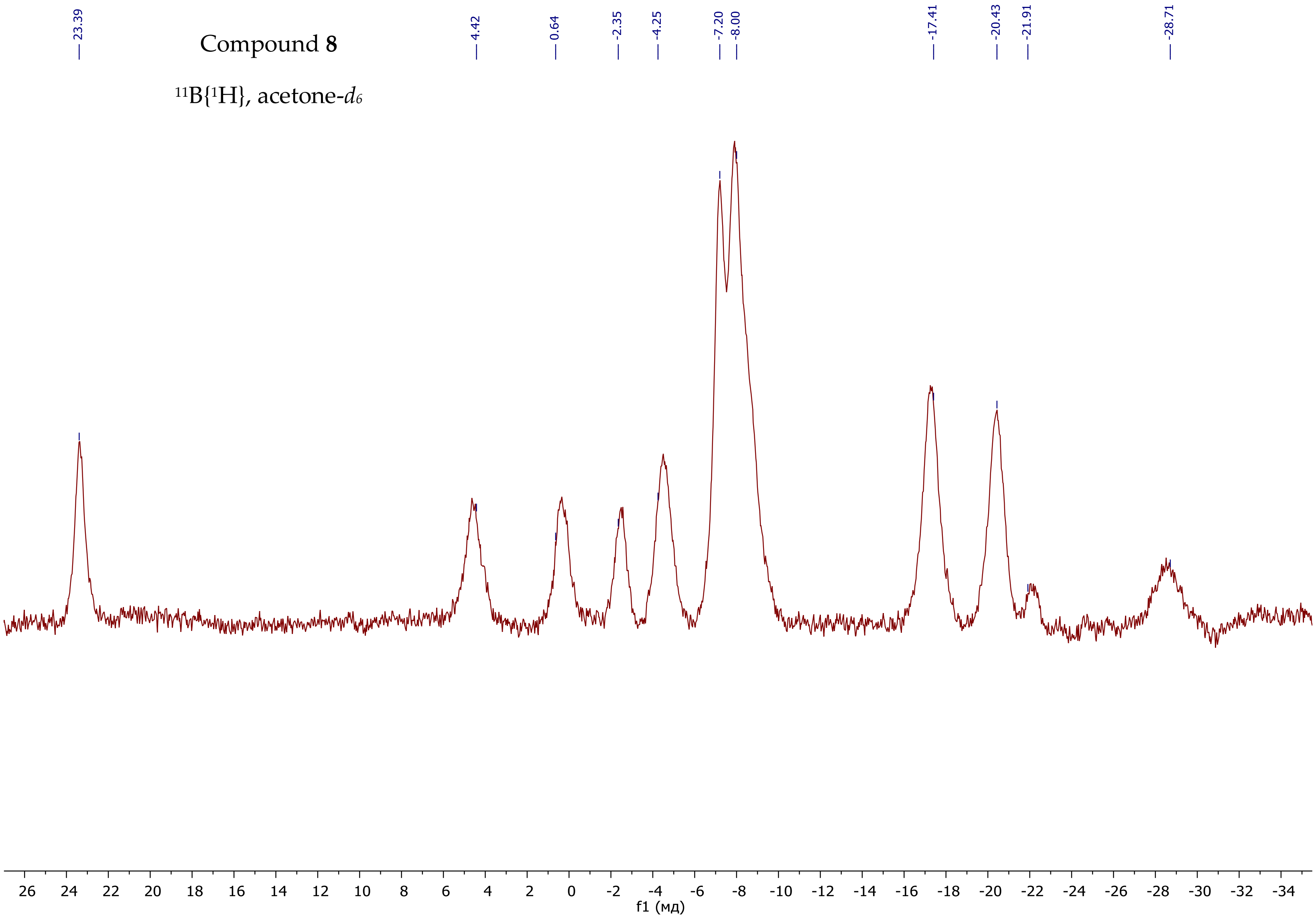

Figure S21. $^{11}B\{^1H\}$ NMR spectrum of compound **8**

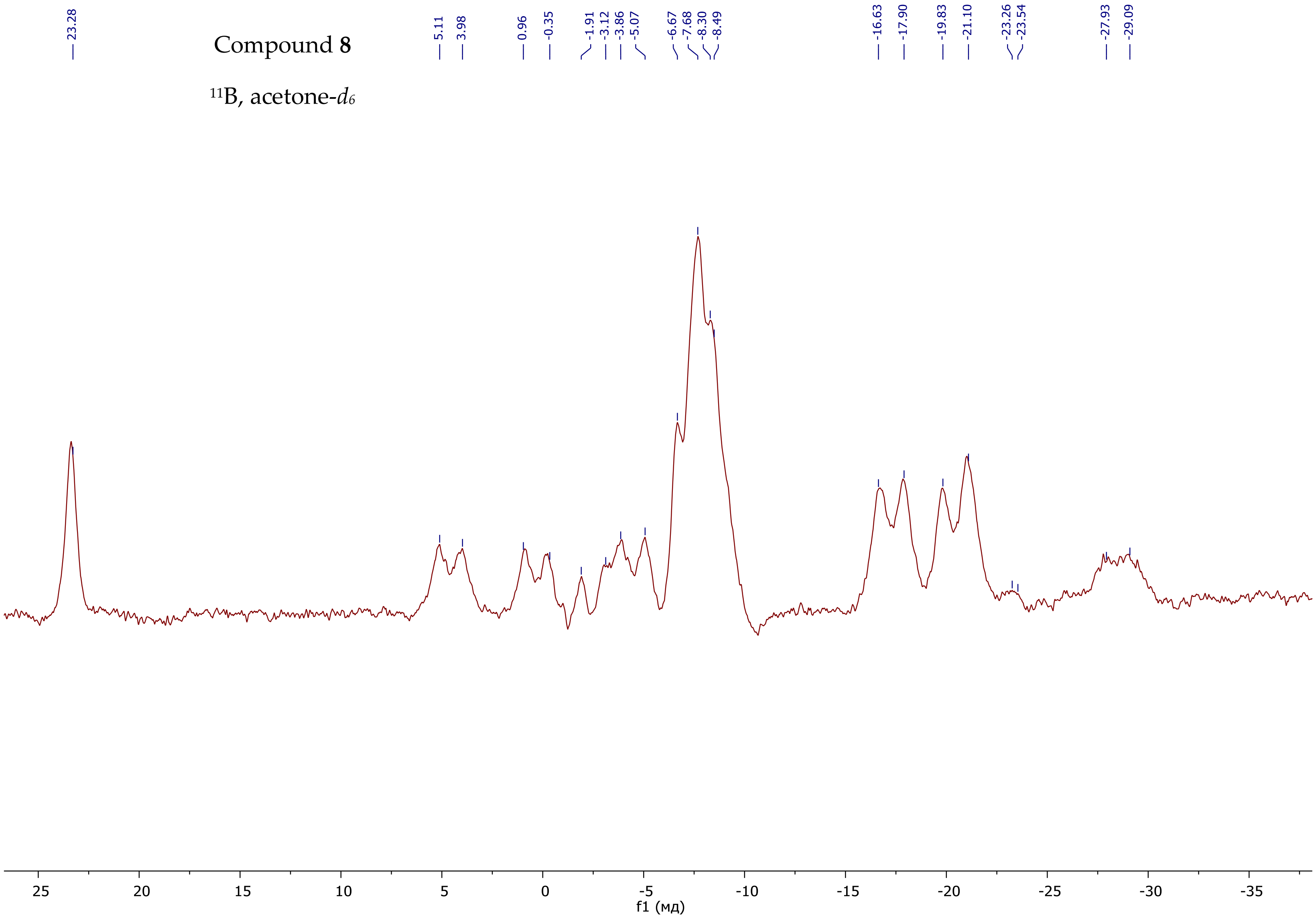

Figure S22. ^{11}B NMR spectrum of compound **8**

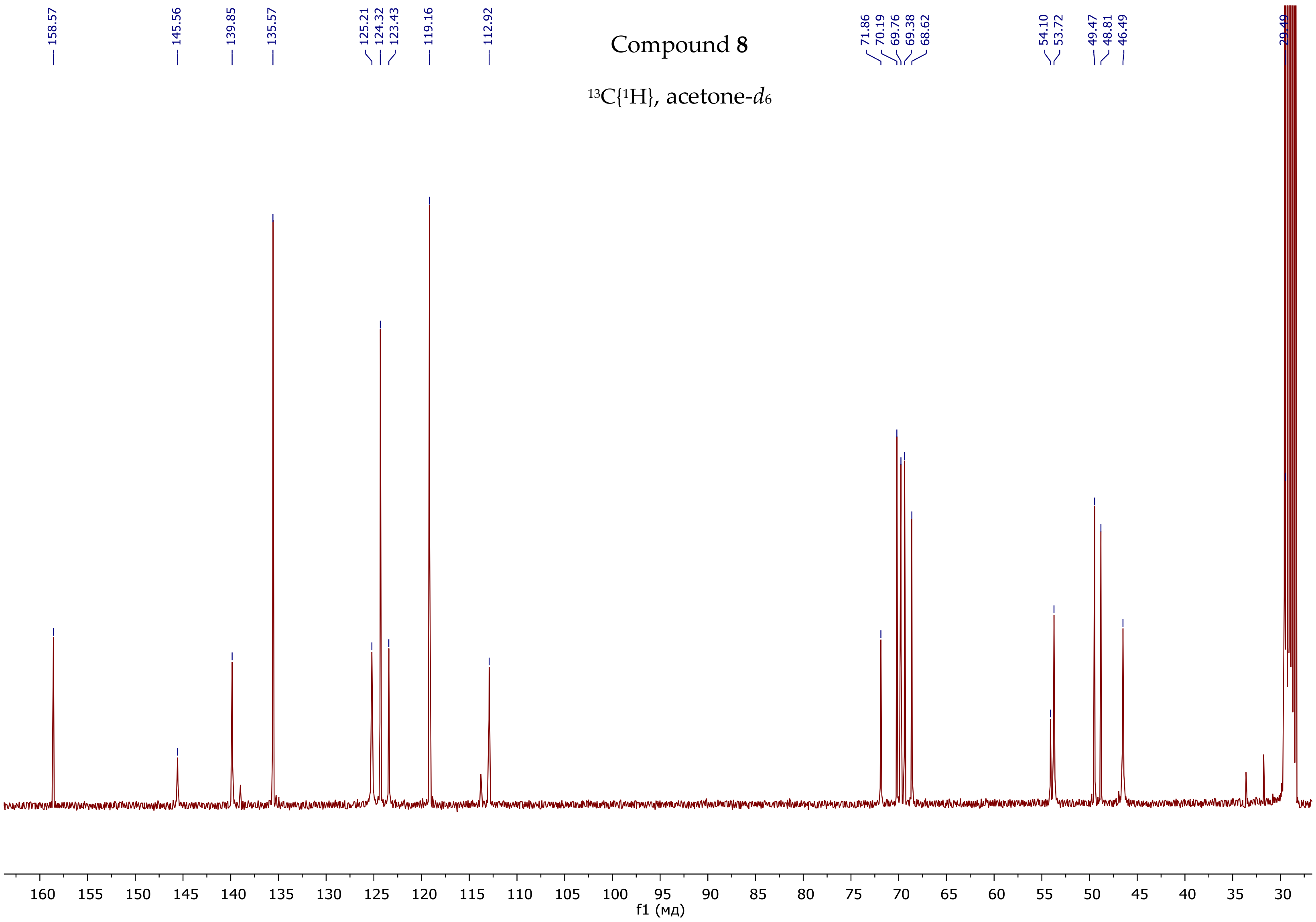

Figure S23. $^{13}C\{^1H\}$ NMR spectrum of compound **8**

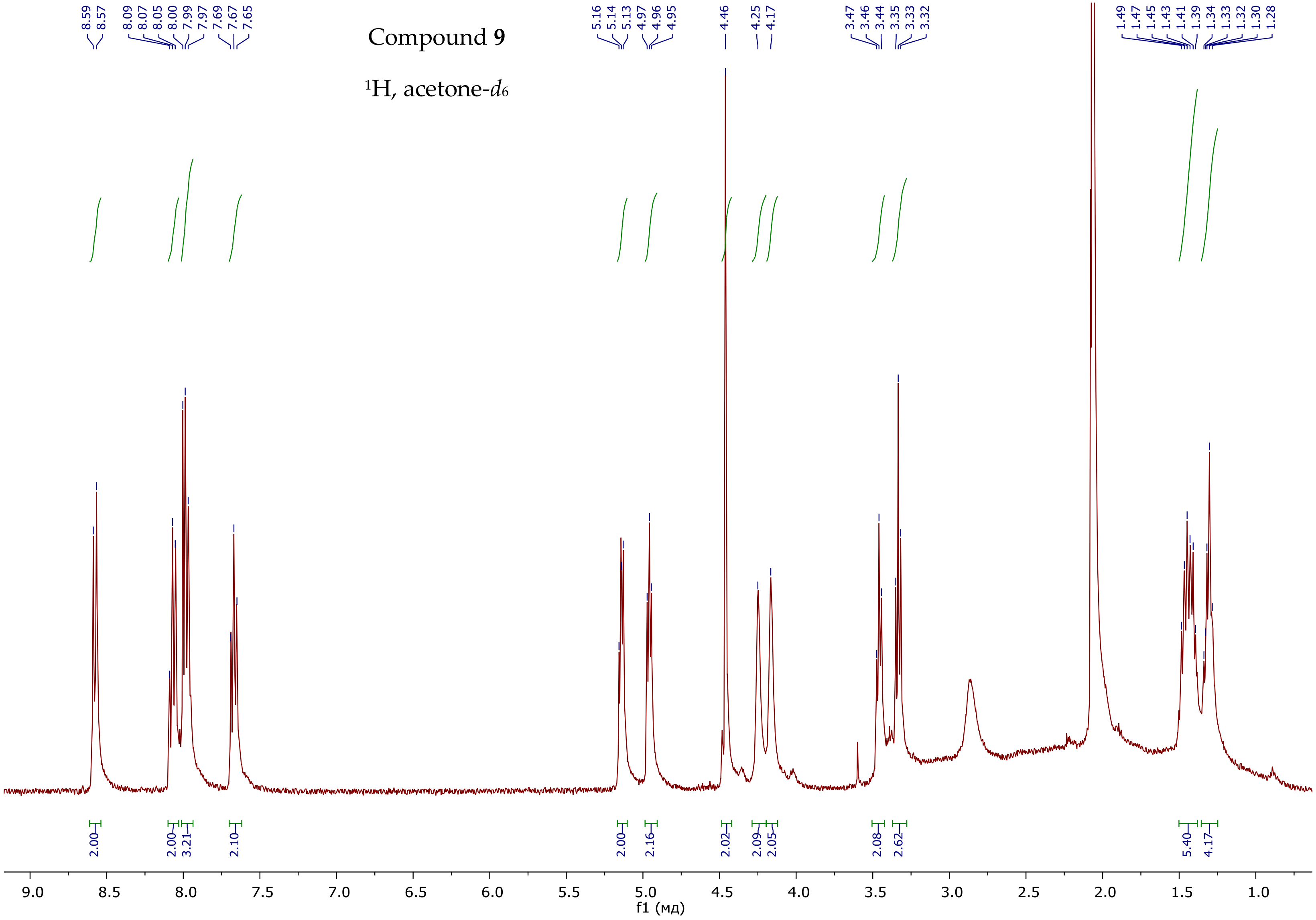

Figure S24. ^{1}H NMR spectrum of compound **9**

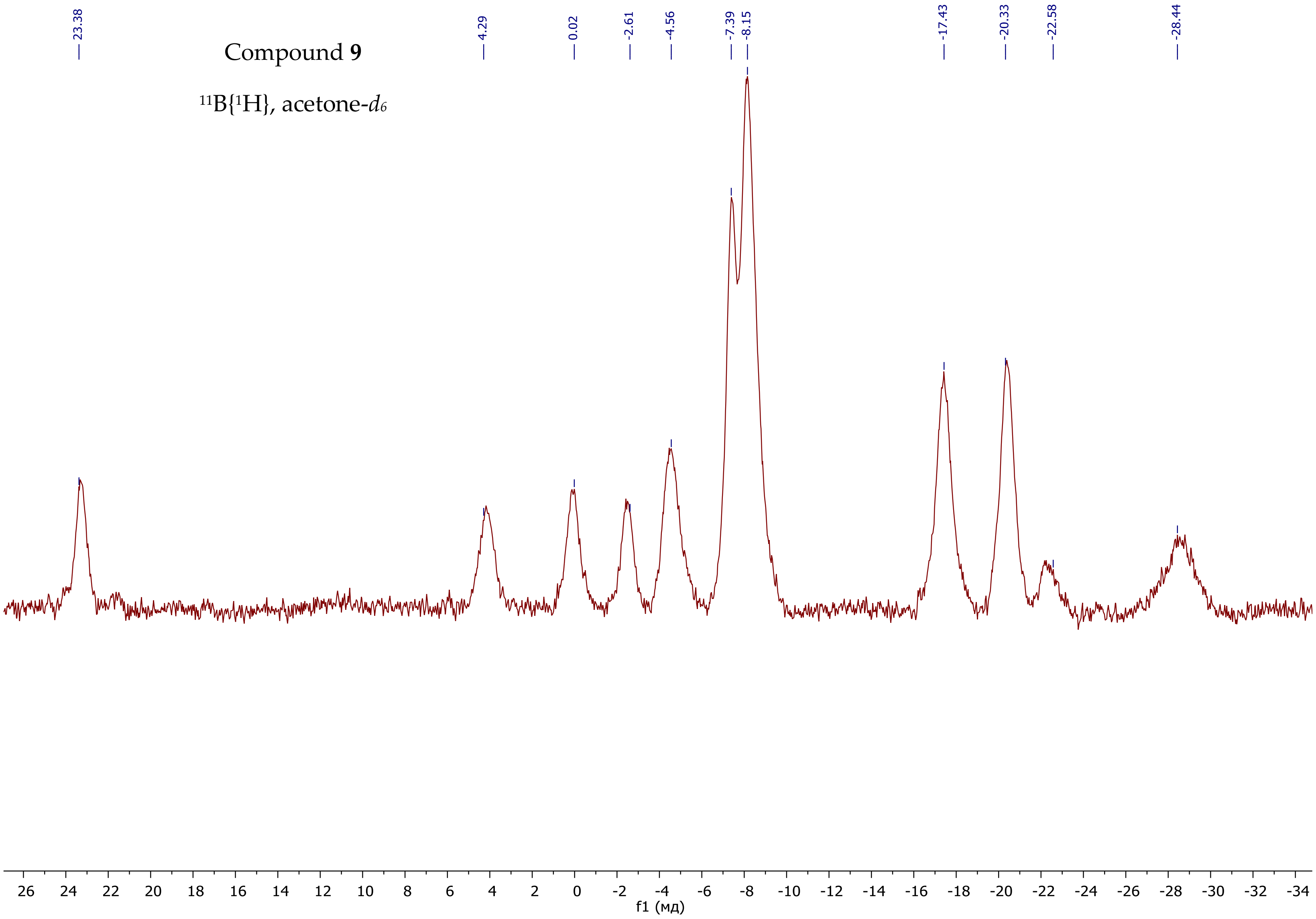

Figure S25. $^{11}B\{^{1}H\}$ NMR spectrum of compound **9**

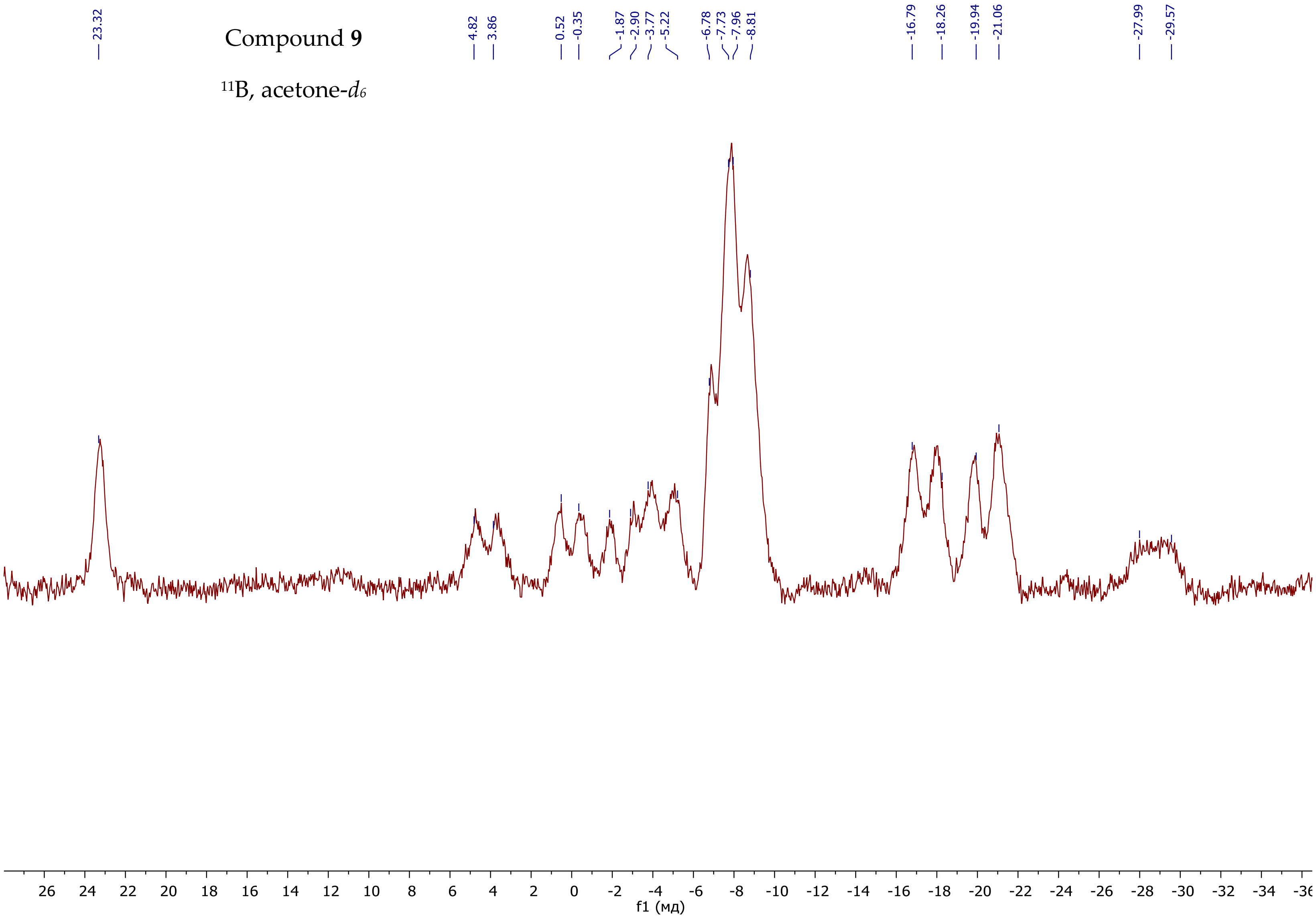

Figure S26. ^{11}B NMR spectrum of compound **9**

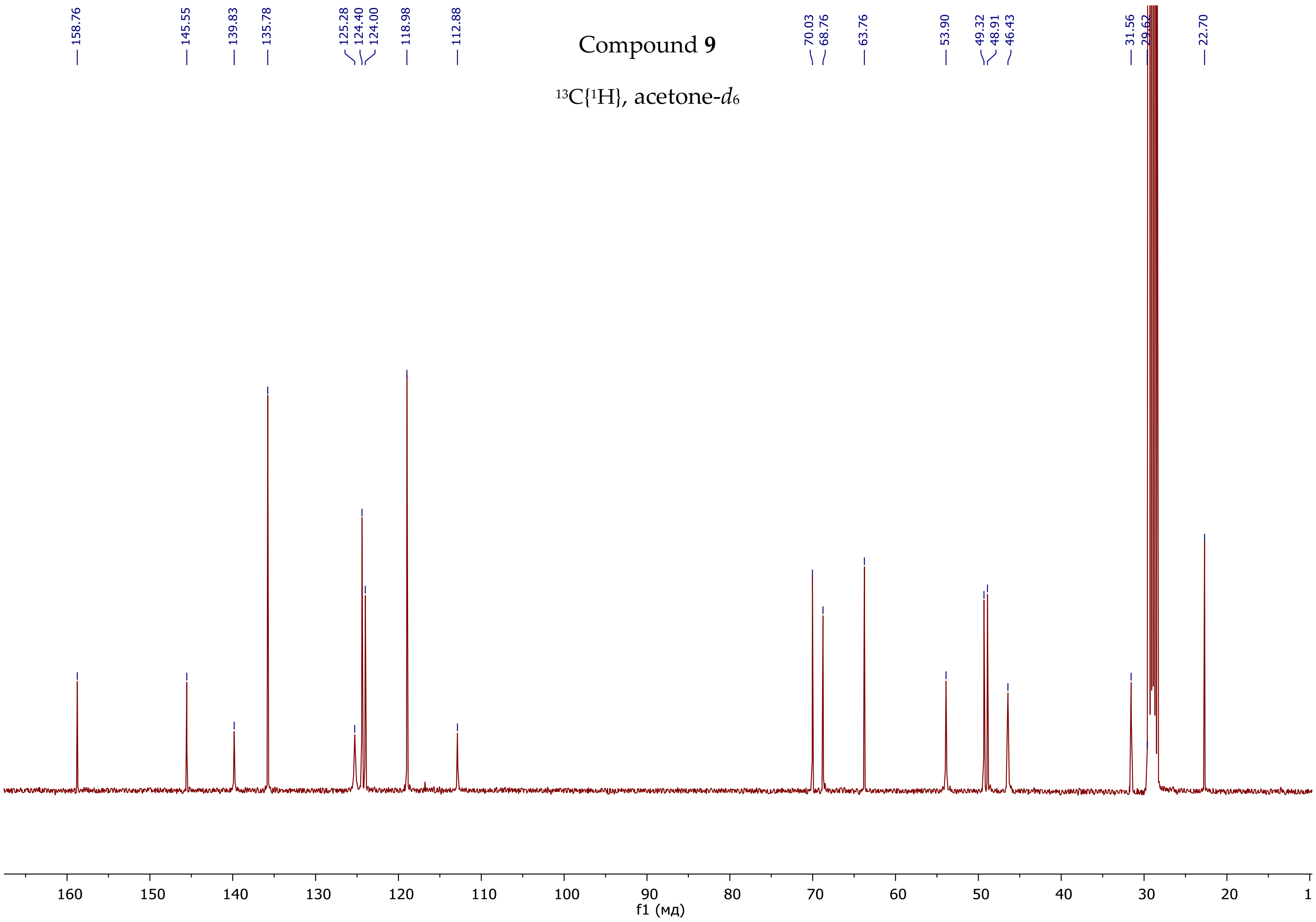

Figure S27. $^{13}C\{^{1}H\}$ NMR spectrum of compound **9**

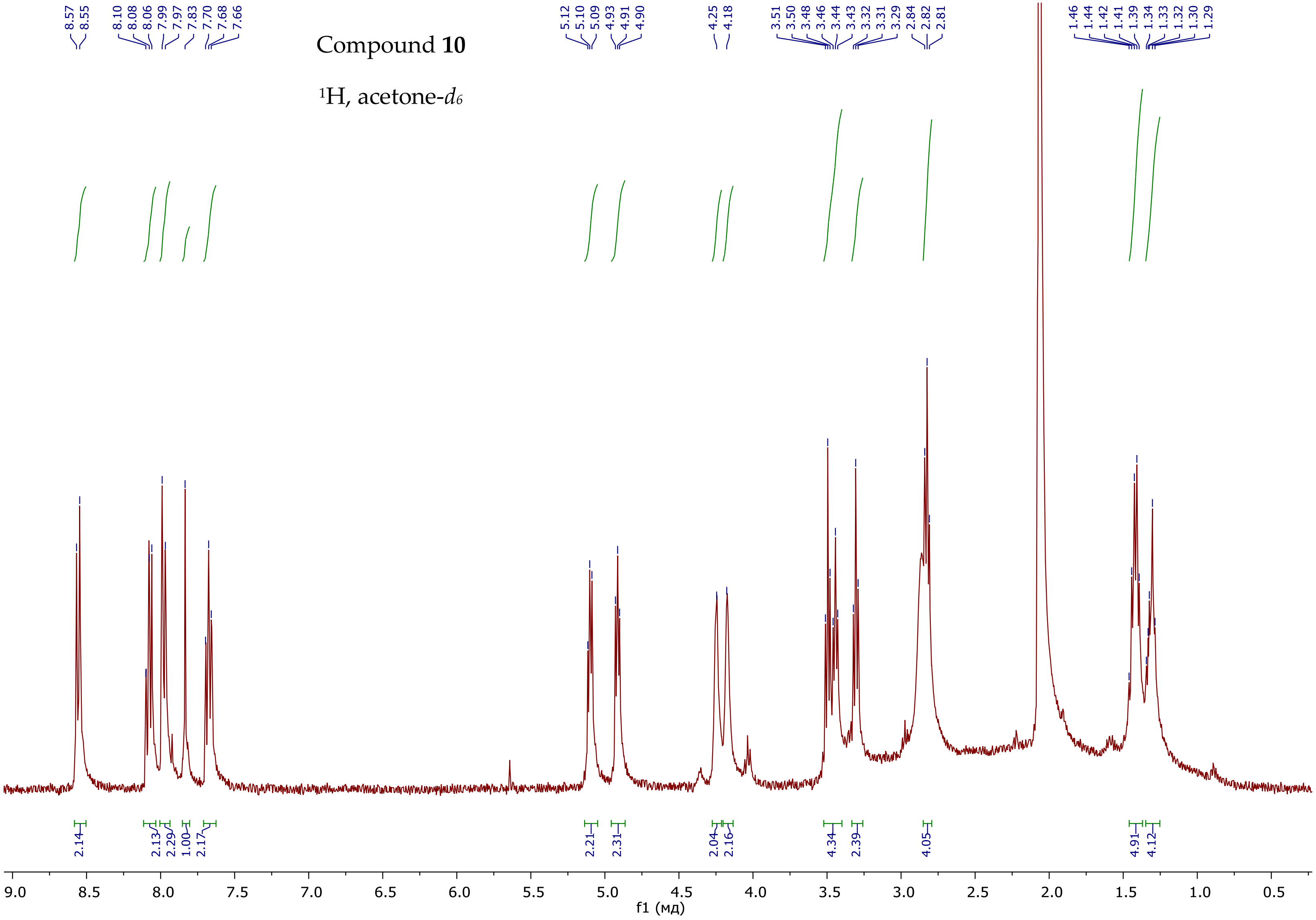

Figure S28. ^{1}H NMR spectrum of compound **10**

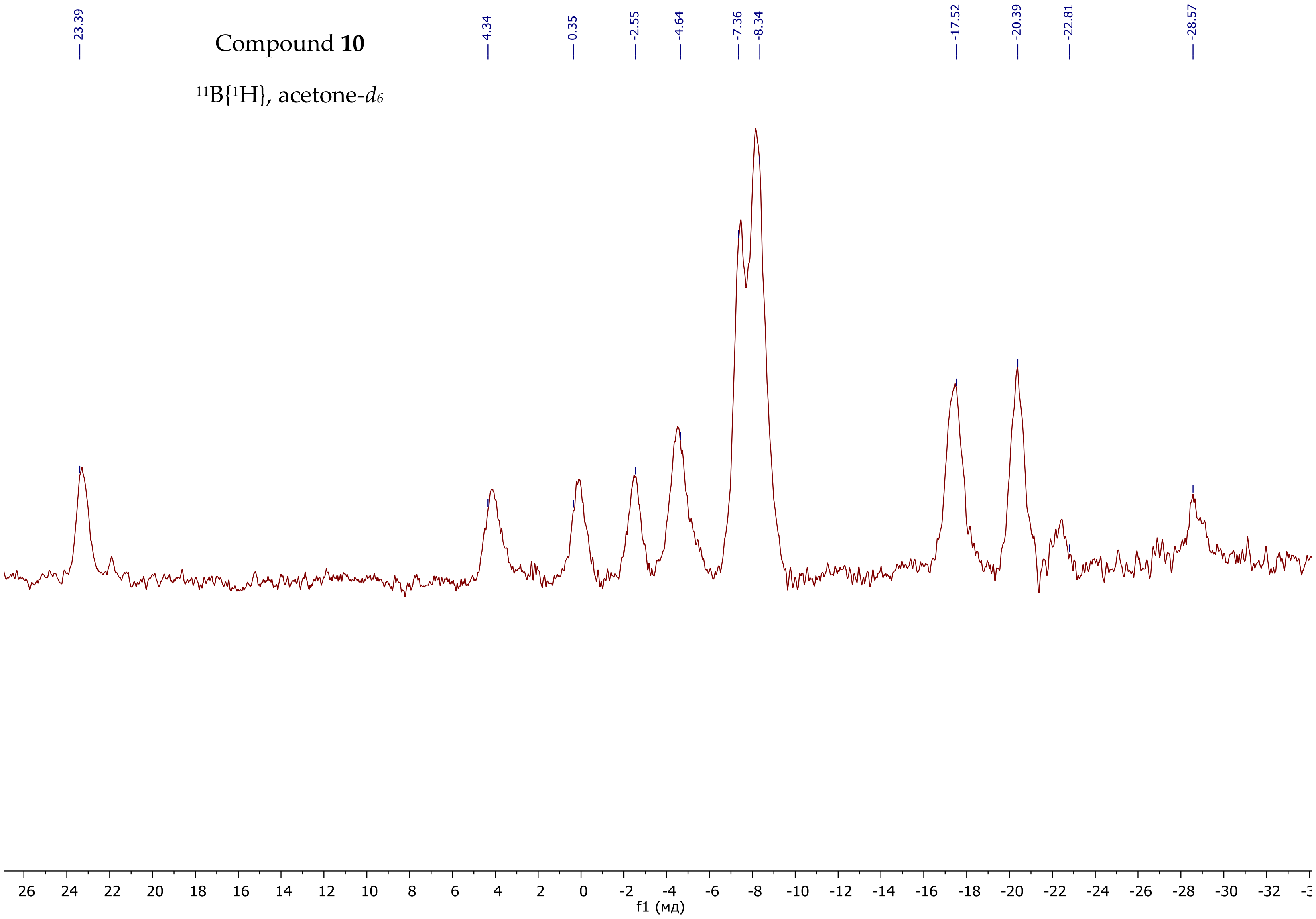

Figure S29. $^{11}B\{^1H\}$ NMR spectrum of compound **10**

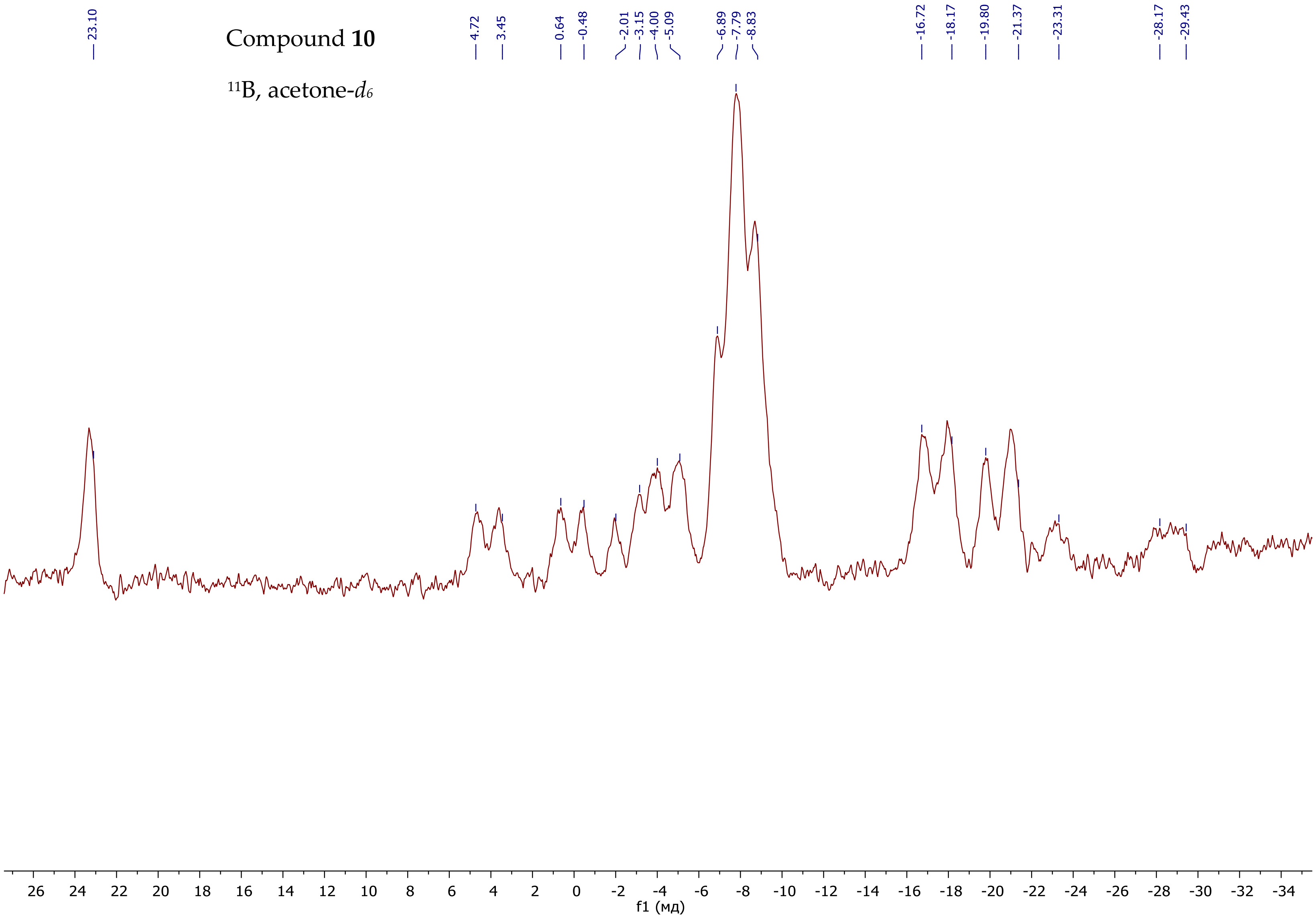

Figure S30. ^{11}B NMR spectrum of compound **10**

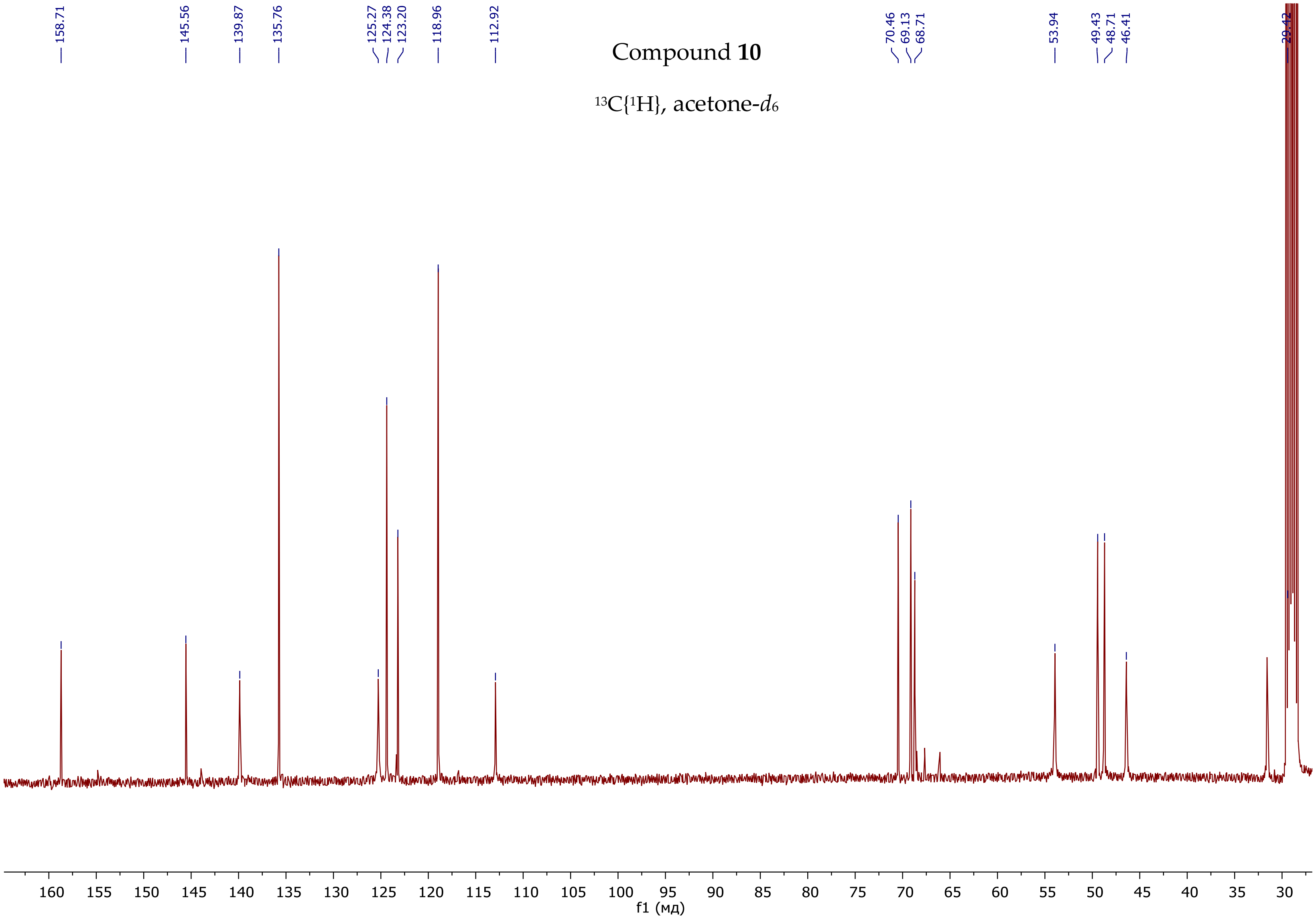

Figure S31. $^{13}C\{^{1}H\}$ NMR spectrum of compound **10**
